# Assessing Flooding from Changes in Extreme Rainfall: Using the Design Rainfall Approach in Hydrologic Modeling

**DOI:** 10.3390/w17152228

**Published:** 2025-07-26

**Authors:** Anna M. Jalowska, Daniel E. Line, Tanya L. Spero, J. Jack Kurki-Fox, Barbara A. Doll, Jared H. Bowden, Geneva M. E. Gray

**Affiliations:** 1 Office of Research and Development, U.S. Environmental Protection Agency, Research Triangle Park, NC 27711, USA; 2 Department of Biological and Agricultural Engineering, North Carolina State University, Raleigh, NC 27695, USA; 3 Department of Applied Ecology, North Carolina State University, Raleigh, NC 27695, USA; 4 Office of Water, U.S. Environmental Protection Agency, Washington, DC 20004, USA

**Keywords:** extreme rainfall, extreme flooding, precipitation intensity–duration–frequency estimates (PIDF), tropical cyclones, design rainfall approach, hydrologic modeling, hydraulic modeling, HEC-HMS, HEC-RAS, future flooding, EPA dynamically downscaled ensemble (EDDE)

## Abstract

Quantifying future changes in extreme events and associated flooding is challenging yet fundamental for stormwater managers. Along the U.S. Atlantic Coast, Eastern North Carolina (ENC) is frequently exposed to catastrophic floods from extreme rainfall that is typically associated with tropical cyclones. This study presents a novel approach that uses rainfall data from five dynamically and statistically downscaled (DD and SD) global climate models under two scenarios to visualize a potential future extent of flooding in ENC. Here, we use DD data (at 36-km grid spacing) to compute future changes in precipitation intensity–duration–frequency (PIDF) curves at the end of the 21st century. These PIDF curves are further applied to observed rainfall from Hurricane Matthew—a landfalling storm that created widespread flooding across ENC in 2016—to project versions of “Matthew 2100” that reflect changes in extreme precipitation under those scenarios. Each Matthew-2100 rainfall distribution was then used in hydrologic models (HEC-HMS and HEC-RAS) to simulate “2100” discharges and flooding extents in the Neuse River Basin (4686 km^2^) in ENC. The results show that DD datasets better represented historical changes in extreme rainfall than SD datasets. The projected changes in ENC rainfall (up to 112%) exceed values published for the U.S. but do not exceed historical values. The peak discharges for Matthew-2100 could increase by 23–69%, with 0.4–3 m increases in water surface elevation and 8–57% increases in flooded area. The projected increases in flooding would threaten people, ecosystems, agriculture, infrastructure, and the economy throughout ENC.

## 1. Introduction

Tropical cyclones (TCs) are the costliest weather and climate disasters in the U.S., predominantly affecting the southeastern states. During 1980–2024, TCs represented 17% of the billion-dollar events across the U.S., but they represented 53% of the cumulative, inflation-adjusted cost [1]. Observations during 1949–2018 revealed that 25% of the top 100 4-day rainfall events nationwide were generated by TCs [2]. Freshwater flooding induced by extreme rainfall associated with TCs (TCFF) is a major threat from TCs. In the U.S., TCFF events were responsible for over one-half of the deaths during 1970–1999 and one-third of the deaths during 1963–2012 that were directly associated with TCs [3]. Flooding from storm surges emerged as the leading cause of TC deaths at the beginning of the 21st century [3,4]. TCFF also triggers infrastructure failure [5], water pollution [6], and socioeconomic struggle in low-lying communities [7] and greatly increased nutrient loading to the coastal ecosystems, inducing positive climate feedback [8,9]. Studies depict a positive correlation between atmospheric warming and the increased risk of flooding in the future due to the intensification of the hydrologic cycle [10]. The hydrologic cycle is projected to intensify alongside future TC rainfall due to the increased water vapor availability, reduction in TC forward speed, and lengthening of the hurricane season [11–15]. Additionally, studies have shown that TCs may increase in frequency, demonstrated through modeling [14] and based on changes in atmospheric drivers like La Niña [15,16]. Subsequently, U.S. built infrastructure that is already experiencing the impacts of changes in the hydrologic cycle will likely be overwhelmed by the projected total volume of water and increased flood risk [17,18]. Thus, quantifying changes in future extreme rainfall, the associated discharge, and spatial extent of the resultant flooding would facilitate necessary changes in rainfall and flood design standards and improve flood control management strategies to increase resilience of local communities and in various sectors, such as transportation and emergency management.

**Figure 1.**
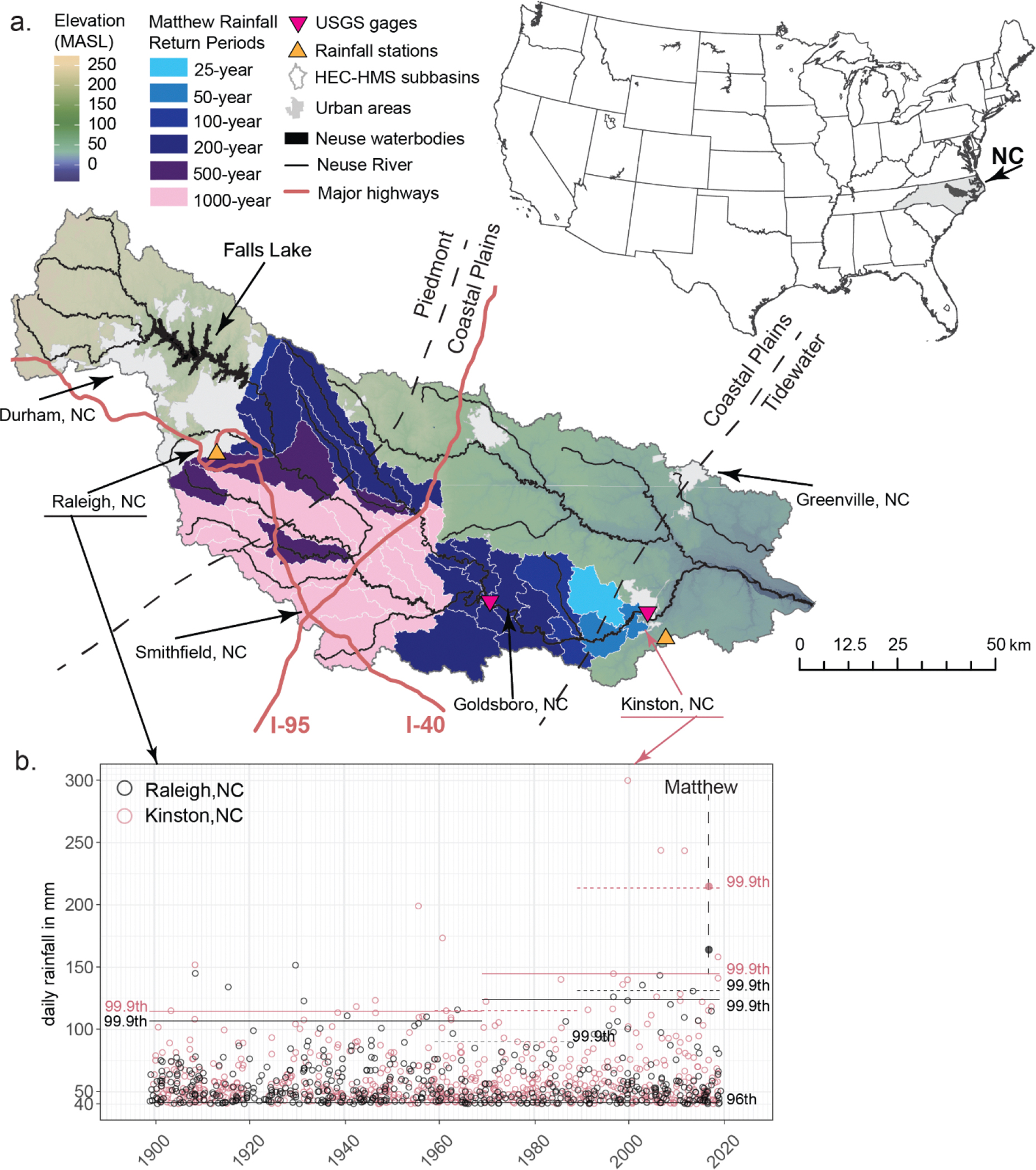
(**a**) The Neuse River watershed and its location within North Carolina and the U.S. Overlaid on the Neuse River watershed map is the NRB and its 44 sub-basins used in HEC-HMS, color-coded by the associated Atlas 14 return period for the total rainfall observed from Matthew. The locations of rainfall measurements at Raleigh, NC, and Kinston, NC, are indicated by yellow triangles (pointing upward), and the USGS stream gages at Goldsboro, NC, and Kinston, NC, are indicated by red triangles (pointing downward). (**b**) Observed extreme daily rainfall events (mm) for Raleigh (black dots) and Kinston (pink dots) exceeding the 96th percentile (as calculated for Raleigh using 1899–2018). The 99.9th percentile thresholds for two 30-year periods (1959–1988 and 1989–2018) are represented by black dashed lines for Raleigh and pink dashed lines for Kinston. The solid black horizontal lines for Raleigh and solid pink lines for Kinston represent 99.9th percentile thresholds for 1969–2018 (50 years) and 1899–1968 (the preceding 70 years of data).

Observations for 1851–2021 show that TCs affected North Carolina, USA (NC; Figure 1) on average 2.2 times a year [19], causing most of the large-scale flooding events in the state [20]. Among the most impactful TCs that affected NC was Hurricane Matthew (“Matthew”), which made landfall in South Carolina on 8 October 2016 as a Category 1 hurricane [21,22] and ranks 72nd in a nationwide analysis of the highest 4-day rainfall events over any area of 50,000 km^2^ during 1949–2018 [2]. The heaviest rainfall occurred in Eastern North Carolina (ENC; 3-day rainfall of 481 mm near Evergreen, NC). Record-breaking rainfall was also recorded in 17 ENC counties, and U.S. Geological Survey (USGS) stream gages recorded new maximum peaks at 26 locations [23]. The highest flood crests were recorded in Neuse River Basin (NRB) in Goldsboro, NC (USGS-02089000 [24]; 9.1 m at 1512 cms) and in Kinston, NC (USGS-02089500 [25]; 8.6 m at 1082 cms) in the aftermath of Matthew, causing unprecedented flooding in NRB (Figure 1a; Figure S1). The NRB headwaters in the Piedmont (in central NC) feed the Falls Lake reservoir (Figure 1a), which provides active flood control for downstream communities. The Neuse River flows through the Piedmont and Coastal Plains to empty into the Pamlico Sound, which is separated from the Atlantic Ocean by a chain of barrier islands (Figure 1a). The Coastal Plains region of ENC is vulnerable to extended flooding due to low elevation [7].

The combination of storm surge inundation and TCFF from Matthew’s excessive rainfall caused 49 deaths, USD 10.9 billion in damages [1], and over 3 million evacuees from coastal areas. In ENC, flooding from Matthew forced closure of parts of two major highways (Interstates 40 and 95; Figure 1) for more than a week, eliminating access to a major coastal shipping port in Wilmington, NC, and disrupting transportation along the U.S. East Coast. Following these disturbances, in 2018, the North Carolina Department of Transportation (NCDOT) and the North Carolina Division of Emergency Management (NCEM) jointly commissioned a study to analyze NRB flooding from Matthew and explore mitigation strategies [26]. In flood risk studies and stormwater management, a common approach [27] is to use a design rainfall/design discharge method with a hydrologic model.

This study proposes a design rainfall approach (DRA) to quantify potential changes in future river discharge and flooding extent within the NRB. Here, the DRA uses the Hydrologic Engineering Center (HEC)–Hydrologic Modeling System (HMS) [28] and the River Analysis System (RAS) [29], a hydraulic model used in the NCDOT and NCEM study, together with DD rainfall data for Matthew from a previous study [30], to quantify potential changes in future river discharge and flooding extent within the NRB (Figure 1a). The DRA localizes future trends in rainfall intensities and can be applied to individual historical storms (e.g., Matthew) to quantify the potential impacts of climate change on flooding events. The methods used in this study consider changes in precipitation, hydrologic, and hydraulic processes and can be used to stress test the watershed system or watershed infrastructure using projected data. This study explored the future changes in both 3-day and 1-day rainfall events from Matthew. Although Matthew was classified as a 3-day storm [2], rainfall over the NRB predominantly occurred in 1 day.

Global climate models (GCMs) provide plausible future scenarios at coarse spatial and temporal scales but cannot represent atmospheric processes at scales that support regional and local decision-making. Regional climate models (RCMs) are used to downscale GCMs to finer resolutions via observation-driven statistical associations (i.e., statistical downscaling; “SD”) or via complex physics-based models (i.e., dynamical downscaling; “DD”). The SD generation requires fewer computational resources and less time and is available at finer spatial resolutions (0.8–6 km) than DD (here, 25 and 36 km), but SD is often limited to daily outputs across a broad ensemble of scenarios. By contrast, DD is computationally expensive, so fewer scenarios can be generated than with SD. However, DD uses scale-appropriate physics, topography, and land use and land cover to create holistic and multivariate representations of the local implications from changing climate. Unlike SD, DD is often available at sub-daily (and sometimes sub-hourly) temporal increments.

This study used five GCMs that were evaluated among 41 members of the Coupled Model Intercomparison Project Phase 5 (CMIP5) ensemble. CMIP6 GCMs became publicly available after the study was completed and thus were not included in this study. Chosen CMIP5 GCMs varied in climatological skill, and, based on mean and extreme statistics, none of these models were outliers [31]: CESM-CCSM4 [32] was ranked 3rd, HAD-GEM2-ES [33] 7th, MPI-ESM-MR [34] 9th, GFDL-CM3 [35] 17th, and GFDL-ESM2M [36,37] 35th within the ensemble. These GCMs produced average biases in mean rainfall over the contiguous United States (CONUS) and even smaller biases for eastern North America, ranging from −8% in winter and +8% in summer (CESM) to less than 0.1% (GFDL-ESM2M and HAD-GEM2-ES) [38]. The GCMs were downscaled using DD and/or SD (Table 1). The five DD GCMs used in this study have comparable methodologies (the same RCM), were available to the authors at the time of this study (Table S2), and provide hourly precipitation data suitable for hydrologic modeling inputs.

This study (1) analyzes historical changes in rainfall characteristics using long-term observations from two stations within NRB (Figure 1) and compares them with the historical period that was modeled by five GCMs and downscaled using DD and SD [39,40]; (2) analyzes changes in future rainfall statistics from five GCMs downscaled using DD and SD [39,40]; and (3) applies a DRA to quantify potential changes in future river discharge and flooding extent within the NRB under those downscaled scenarios (Figure 1a).

## 2. Materials and Methods

### 2.1. Historical Observed Rainfall and Flow Data

The 120-year daily rainfall datasets for stations “Raleigh State Univ, NC” and “Raleigh AP” for 1899–2018 and “Kinston 7SE, NC” and “Kinston AG Research, NC” for 1899–2018 (Figure 1b) [41] were acquired from http://scacis.rcc-acis.org/ (accessed on 11 June 2025). Extreme rainfall thresholds were calculated from the entire dataset at each station for percentile ranks of 96th, 98th, 99th, 99.5th, 99.8th, and 99.9th of the normal distribution. The change (i.e., delta or Δ) in daily rainfall intensity thresholds between two historical periods were developed using four subsets of the datasets in Raleigh and in Kinston for 70- and 50-year (uneven) periods (1899–1968 and 1969–2018; Figure 1b) and for two 30-year (even) periods (1959–1988 and 1989–2018). This study used 30-year or longer periods to reflect general climatological standards in data record length [42].

Rainfall frequency estimates represent the probability (return period) that rainfall of a given intensity (rainfall accumulation per unit time) or depth (rainfall accumulation) will occur over a given duration (period of rainfall accumulation). Rainfall frequency estimates based on observations were obtained from NOAA Atlas 14 [43], which was also used to create a temporal 2nd-quartile storm distribution (Figure S3).

NRB discharge data were obtained for USGS stream gages in Goldsboro and in Kinston (USGS-02089000 [24] and USGS-02089500 [25], respectively). The channel bathymetry was based on the cross sections in the Flood Risk Information System (FRIS) from the North Carolina Floodplain Mapping Program [44]. The FRIS was also a source of spatial data delineating extents of 100-year and 500-year floods (1% and 0.2% annual-chance of flooding floodplains, respectively), based on Federal Emergency Management Agency (FEMA) flood maps. The FEMA maintains and updates flood maps based on the best available data, including, but not limited to, topography, river cross sections, river flow, water controlling structures, hydrologic, and hydraulic modeling.

### 2.2. Modeled Historical and Future Rainfall

Future rainfall data were subset from six DD simulations from the CMIP5 ensemble (Table 1) [38] under two Representative Concentration Pathways (RCPs) [45]: one under RCP4.5 [46] and five under RCP8.5 [47] that were generated by U.S. Environmental Protection Agency (EPA) as the EPA Dynamically Downscaled Ensemble (EDDE) [48,49] version 1 (“DD-EDDE”) and from the North American Coordinated Regional Downscaling Experiment (NA CORDEX; “DD-CDX” [50]). CMIP6 GCMs became publicly available after the study was completed, so CMIP6 scenarios are not included here.

All DD simulations (Table S2) were generated using the Weather Research and Forecasting (WRF) model [51], and DD hourly output was not bias corrected. This study used relative change in rainfall thresholds instead of explicitly modeled rainfall to facilitate comparison between bias-corrected SD datasets with non-bias-corrected DD datasets, as well as between different horizontal grid spacings used by each data developer. Additionally, comparison using Δ de-emphasizes individual GCM biases [30]. The DD-EDDE consists of two GCMs downscaled to 36 km grid spacing: CESM-CCSM4 [32] covering 1975–2005 and 2025–2099 (“CESM-4.5” and “CESM-8.5”), and GFDL-CM3 [35] covering 1995–2005 and 2025–2099 (“GFDL-CM3–8.5”). The WRF model configurations used in DD-EDDE were validated against historical data in previous studies [52–59] and were used in the Fourth National Climate Assessment [60] and elsewhere [30,52]. The DD-CDX consists of three GCMs under RCP8.5: “HAD-GEM2-ES-8.5”, “MPI-ESM-MR-8.5”, and “GFDL-ESM2M-8.5” [50] for 1950–2005 and 2006–2099. The choice of the GCMs for this study was driven by the availability of the DD hourly precipitation data at the time of the study and by the common downscaled method (WRF).

Additionally, the study used two bias-corrected datasets generated using two SD methods from the five corresponding CMIP5 GCMs for 1950–2005 and 2006–2099 under RCP8.5 (Table 1): Localized Constructed Analogs (“SD-LOCA”) [61,62] and Multivariate Adaptive Constructed Analogs (“SD-MACA”; MACAv2-METDATA) [63,64]. The daily SD data were not used in hydrologic modeling due to coarse temporal resolution.

### 2.3. HEC-HMS Model Development and Calibration

HEC-HMS version 4.2.1 [28] was used to simulate runoff processes in the NRB (4686 km^2^) using inputs derived from rainfall, topography, stream/river channel, soil, and land cover data ([26], Supplementary Material). HEC-HMS addresses variations in landscape characteristics by dividing the basin of interest into sub-basins (43 in NRB) connected in a dendritic network (237.5 km of primary streams and 486 km of secondary streams in NRB), defining the physical attributes of the sub-basins, and representing spatial and temporal characteristics of the rainfall. Detailed parameterization in using the HEC-HMS model for Matthew is in the NCDOT NCEM study report ([26], Supplementary Material).

Although Matthew lasted for 3 days in the region, rainfall over NRB predominantly occurred in 1 day. Therefore, in the NCDOT NCEM study, HEC-HMS was calibrated using 1-day rainfall data [26]. The temporal storm distribution of rainfall was guided by an Atlas 14 [43] 2nd-quartile storm and compared with observations [26]. Incremental rainfall depths for 50% probability from the 2nd-quartile 1-day storm [43] (Figure S3) were used in the HEC-HMS model for each of 44 sub-basins of the NRB to create hydrographs.

The modeled hydrographs for the Neuse River at Goldsboro and at Kinston (Figure 1) for Matthew were compared to the observed discharges recorded at the USGS stream gages. HEC-HMS was calibrated by adjusting curve numbers, lag times, and channel routing parameters and validated using a coefficient of determination (R^2^) and the Nash–Sutcliffe model efficiency coefficient (NSE) [65]. At Goldsboro, a “very good” agreement was achieved in the rising limb (R^2^ = 0.99) and in the falling limb (R^2^ = 0.98) of the hydrograph [65]. At Kinston, there was a very good agreement between the simulated and observed hydrographs (R^2^ = 1.00) [65]. The modeled peak discharge was within 1% of the observed for both gaging stations. At Goldsboro, the observed peak of 1512 cms was modeled as 1504 cms, and at Kinston, the observed and modeled peaks were at 1082 cms. Further, the NSE was greater than 0.96 for both stations, which also indicates very good agreement with the observations [65]. These data indicate very good agreement between modeled and observed discharges, reflecting optimal parameterization of the NRB hydrologic model.

### 2.4. HEC-RAS Model Development and Calibration

Two-dimensional HEC-RAS (v6.2) models [29,66] were developed for ~16 km sections (model area of ~110 km^2^) of the Neuse River in Goldsboro (reach length 6.6 km) and Kinston (reach length 4.6 km). The model grid spacing was set at 76.2 m and refined to 15.2 m along the river channel and at major road embankments. Elevations for the model grid were based on LiDAR-derived 1.524 m resolution digital elevation models [67] for the floodplain and upland. The channel bathymetry was based on the cross sections in the FRIS 1-D HEC-RAS models that were used to develop floodplain maps [44]. Manning’s roughness values were set to vary spatially according to the 2016 National Land Cover Database raster data (30 m resolution) [68], following published guidance [66,69]. Manning’s roughness for the channel was defined by manually tracing the river channel. The downstream boundary condition was set to normal depth with the slope set to the downstream longitudinal slope.

The models were calibrated to the peak discharge recorded at the same USGS stream gages used in HEC-HMS and high-water marks. Manning’s roughness was adjusted until the simulated water surface elevations closely matched the recorded high-water marks. For Kinston stations, the flood high-water marks were collected by the City of Kinston Engineering Department and, for Goldsboro, by the USGS [70]. The calibrated model simulated water surface elevations (WSEs) were within −0.12–0.89% of the recorded high-water marks at Kinston and within −1.25–0.81% at Goldsboro. While the differences between the simulated and observed WSEs were greater at Goldsboro, there is likely more uncertainty associated with these observations, as they were recorded after the storm using debris and water lines; the high-water observations at Kinston were recorded during the flood event.

### 2.5. Design Rainfall Approach

The future runoff was simulated using projected changes in rainfall from three DD-EDDE scenarios, “CESM-4.5”, “CESM-8.5”, and “GFDL-CM3–8.5”, which were created in a previous study [30] based on hourly rainfall (>1 mm), where the future rainfall frequency estimates were derived for each modeled grid cell from annual maximum series (AMS) of the highest annual rainfall accumulation over the study period for 1- and 3-day durations. The 36 km horizontal grid spacing used here can adequately simulate historical precipitation intensity–duration–frequency (PIDF) curves for these durations [30,71]. The AMS of each duration was divided into two 30-year periods: p1 (2025–2054) and p2 (2070–2099). The AMS in each period was fit with a generalized extreme value (GEV) distribution using regional frequency analysis (RFA) based on L-moments using the R package “lmomRFA” Version 3.3 [72,73]. Each modeled grid cell represents an individual site in the RFA. Grid cells were grouped into RFA regions and tested for homogeneity and discordancy [30]. These rainfall frequency estimates (2-, 5-, 10-, 25-, 50-, 100-, 200-, 500-, and 1000-year) were used to calculate Δ between p1 and p2 for each return period (RP) (Table S4) [30]. The Δ represents the projected increase in rainfall intensities by the end of the 21st century (“2100”). For this study, the ENC calculations from Jalowska et al. (2021) [30] were further subset to the NRB region (six grid cells; Figure S3).

The 44 HEC-HMS sub-basins of the NRB were allocated to the six corresponding DD-EDDE grid cells by their location, where >50% of the sub-basin area fell into a given grid cell. Then, the observed Matthew rainfall in each sub-basin was assigned the RP from NOAA Atlas 14 [43] based on its location. Finally, the sub-basin rainfall was adjusted by Δ using the DRA [30]. The procedure was repeated for three future scenarios at two durations, yielding six possible future realizations of Matthew “2100” (“Matthew 2100”; Figure 1; Table S3). Rainfall from each outcome was supplied to the calibrated HEC-HMS to simulate projected discharge of the Neuse River from Matthew 2100.

## 3. Results

### 3.1. Historical Changes in Rainfall Characteristics

The 120-year-long timeseries (1899–2018) of rainfall records [41] from two stations in NRB in Raleigh and Kinston (Figure 1a) were analyzed for the most extreme events, exceeding the 99.9th percentile (top 0.1% or 1‰ of the events; Figure 1b). In Kinston, 75% (18 of 24 records) of the most extreme daily rainfall in the last 120 years were associated with TCs, compared with 42% in Raleigh (Figure 1b; Table S1), which is much farther inland than Kinston (Figure 1). This disparity between the stations emphasizes the increased exposure of ENC communities and infrastructure to frequent TC impacts and excessive flooding and the gradient of the impact inland from the coast. The daily rainfall from Matthew was among the highest recorded at Kinston, and it was the highest recorded at Raleigh (1899–2018; Figure 1b). In the NRB, 39% of the recorded rainfall totals had 1000-year RPs, and an additional 13% of the study area recorded 500-year rainfall (Figure 1a) [43].

The changes in the most extreme observed rainfall events (99.9th percentile; Figure 1b) are driven by the length of the chosen observation record and characteristics of those records (Figure 1b). The changes derived from longer, uneven data records are 16% in Raleigh (1899–1968 to 1969–2018) and 26% in Kinston (1899–1968 to 1969–2018). Changes derived from more recent, 30-year periods (1959–1988 to 1989–2018) are much higher (46% in Raleigh, 86% in Kinston) than for the longer, uneven data records.

**Figure 2.**
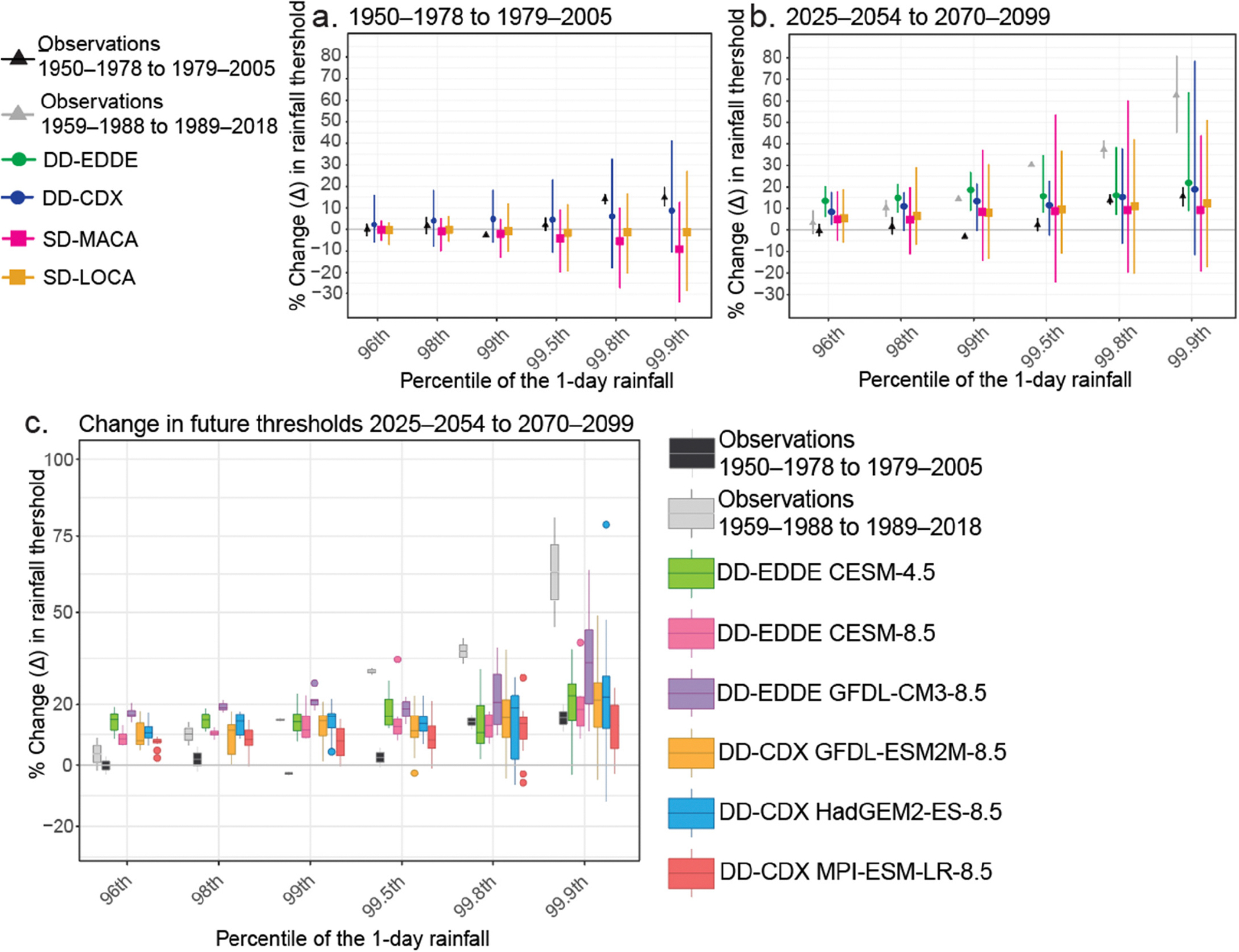
Comparison of DD and SD datasets for the NRB (all grid cells) with station observations from Kinston and Raleigh. (**a**) Comparison of the median Δ in rainfall intensity thresholds between 1950–1978 and 1979–2005 for DD and SD datasets. Note that the simulated historical period is not long enough in DD-EDDE to be included in this comparison. (**b**) Comparison of the median Δ in rainfall intensity thresholds from DD and SD datasets under RCP8.5 by dataset type from 2025–2054 to 2070–2099 and two historical periods: 1950–1978 to 1979–2005 (black) and 1959–1988 to 1989–2018 (gray). (**c**) Comparison of the median Δ in rainfall intensity thresholds for DD models from 2025–2054 to 2070–2099. Changes between observational periods (with colors as in panel **b**) are shown to provide additional context.

Comparisons of the observed Δ in rainfall thresholds between 1950–1978 and 1979–2005 were first examined in the 96th, 98th, 99th, 99.5th, 99.8th, and 99.9th percentiles (Figure 2). SD models best represented the observed Δ between these historical periods for the 96th, 98th, and 99th percentiles (Figure 2a), largely because SD datasets were bias corrected toward the observational records. The positive Δ in the most extreme intensities (>99th percentile) were best represented by DD-CDX models, while SD datasets produced negative Δ for these ranks (Figure 2a). The DD-CDX minima also produced some small, negative Δ in some of the models (Figure 2a), which may reflect that some localized extremes may be suppressed by the more modest grid spacing in the DD data compared with the SD data.

### 3.2. Future Changes in Rainfall Characteristics

In the comparison of future Δ (2070–2099 to 2025–2054; Figure 2b), the DD-EDDE simulations depicted the largest median Δ of all datasets and positive Δ in most percentiles in DD-EDDE and up to the 99th percentile in DD-CDX. DD-CDX produced some negative Δ in the 99th, 99.5th, 99.8th, and 99.9th percentiles but also produced the highest Δ maxima in the 99.9th percentile. Maximum Δ from DD datasets at 99.8th and 99.9th did not exceed observed historical Δ in the NRB for 1959–1988 to 1989–2018. Both DD datasets produced similar median Δ, exceeding median Δ from SD datasets. DD-CDX produced a larger range of Δ than DD-EDDE and a higher median Δ than SD. SD has a comparatively higher range of Δ between models and grid cells than DD. Among the DD models (Figure 2c), all changes in future thresholds from 2025–2054 to 2070–2099 were positive in the 96th and 98th percentiles. DD-EDDE-GFDL-CM3–8.5 produced the highest median Δ for all percentiles and the highest Δ maxima for the 96th, 98th, 99th, and 99.8th percentiles. DD-CDX-HAD-GEM2-ES-8.5 produced both the highest Δ maxima and the lowest negative Δ for the 99.9th percentile.

### 3.3. Future Rainfall from Matthew 2100 Under the Design Rainfall Approach

**Figure 3.**
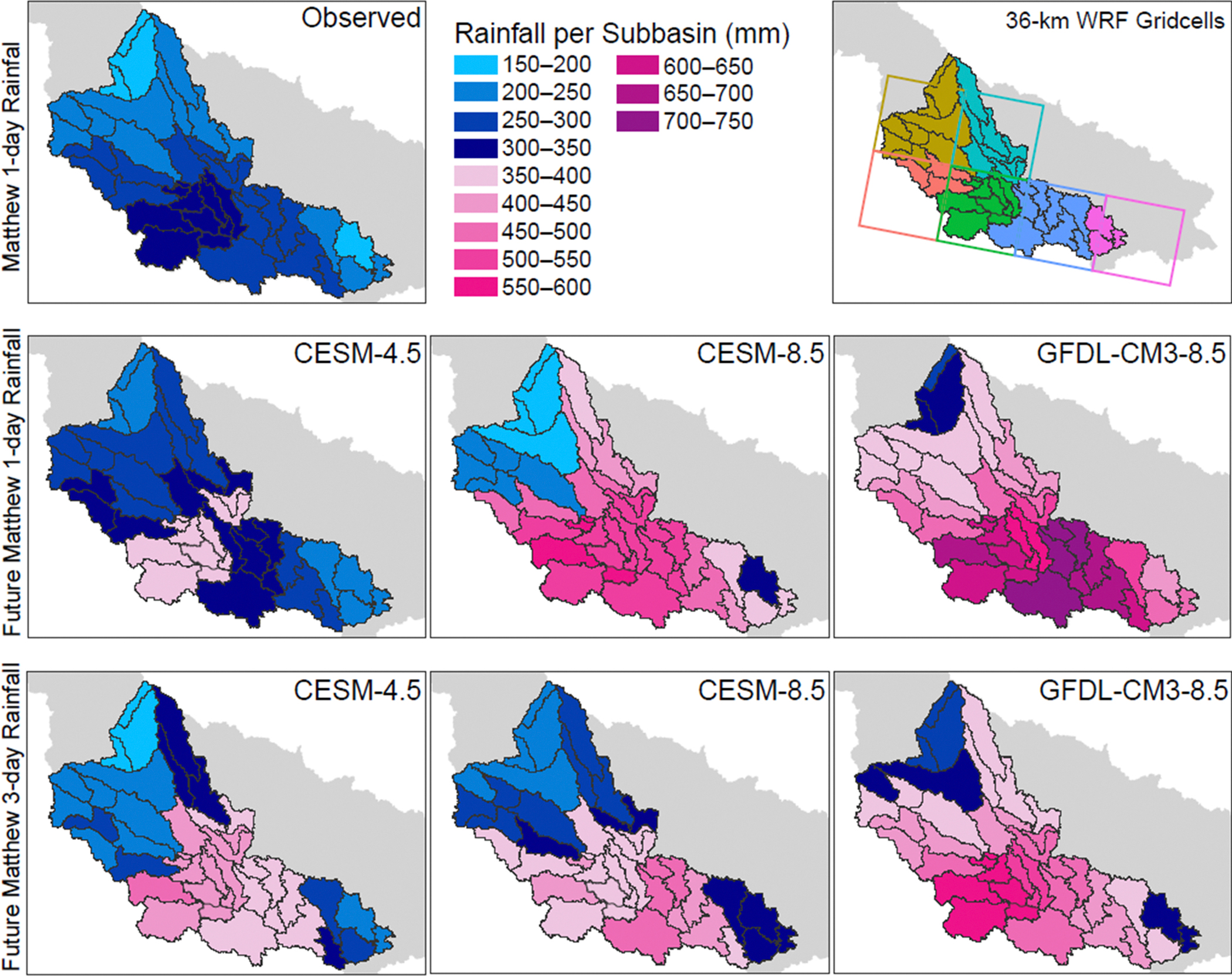
The 44 sub-basins in the NRB, shown with rainfall values used in HEC-HMS modeling. The change from blue to pink in the color scale occurs at 350 mm, which corresponds to the mean 1000-year rainfall in the NRB for a 1-day storm from NOAA Atlas 14. In top, right insert subwatershed colors correspond to WRF grid cells colors the sub-basins were assigned to.

The DRA results derived from three DD-EDDE scenarios [30] were used in HEC-HMS to simulate future Matthew discharge in the NRB. In the DRA, the observed rainfall over a given duration is assigned a corresponding RP from Atlas 14 [43] and then adjusted for Δ in rainfall frequencies that were derived from two projected periods (2025–2054 to 2070–2100). The most rainfall observed from Matthew (Figures 1a and 3) was 337 mm in the central NRB, which falls within confidence intervals of a 1000-year rainfall event. The least rainfall observed in the NRB from Matthew (185 mm) occurred in the northern and eastern parts of the NRB.

Projected rainfall in the NRB from under CESM-4.5 shows higher relative increases in 3-day rainfall than in 1-day rainfall. The 1-day rainfall from the CESM-4.5 scenario increases by 2–28% across all sub-basins (maximum rainfall: 386 mm). The highest Δ were modeled for sub-basins with observed 200-year rainfall from Matthew. In the western sub-basins, the 3-day rainfall from CESM-4.5 increased by 15%, but in central sub-basins with observed 1000-year rainfall from Matthew, it increased as much as 46% in central sub-basins with the 1000-year rainfall, resulting in the maximum rainfall reaching 454 mm (Figure 3; Table S3).

Conversely, in both RCP8.5 scenarios, the projected rainfall Δ is smaller for the 3-day rainfall than for the 1-day rainfall, suggesting higher increases in the shorter-duration rainfall, under RCP8.5. Additionally, 1-day rainfall from downscaled CESM-8.5 shows a larger range in the future Δ, decreasing by up to 16% in western sub-basins and increasing by 40–89% elsewhere, with a maximum rainfall of 579 mm in the sub-basin associated with an observed 100-year rainfall from Matthew. The Δ for 3-day rainfall from downscaled CESM-8.5 increases by less than the 1-day Δ, up to 49% in the sub-basin with a 200-year storm, with the smallest increases in the western sub-basins and with maximum rainfall reaching 456 mm (Figure 3; Table S3). The 1-day GFDL-CM3–8.5 shows the highest Δ (39–112%), with most significant increases in the eastern sub-basins with a 200-year rainfall, and maximum rainfall reaching 716 mm. The 3-day rainfall under GFDL-CM3–8.5 increases by 33–82%; the maximum rainfall in the sub-basin is 596 mm in the sub-basin with observed 1000-year rainfall from Matthew (Figure 3; Table S3).

### 3.4. Future Discharge and Flooding from Matthew 2100

**Figure 4.**
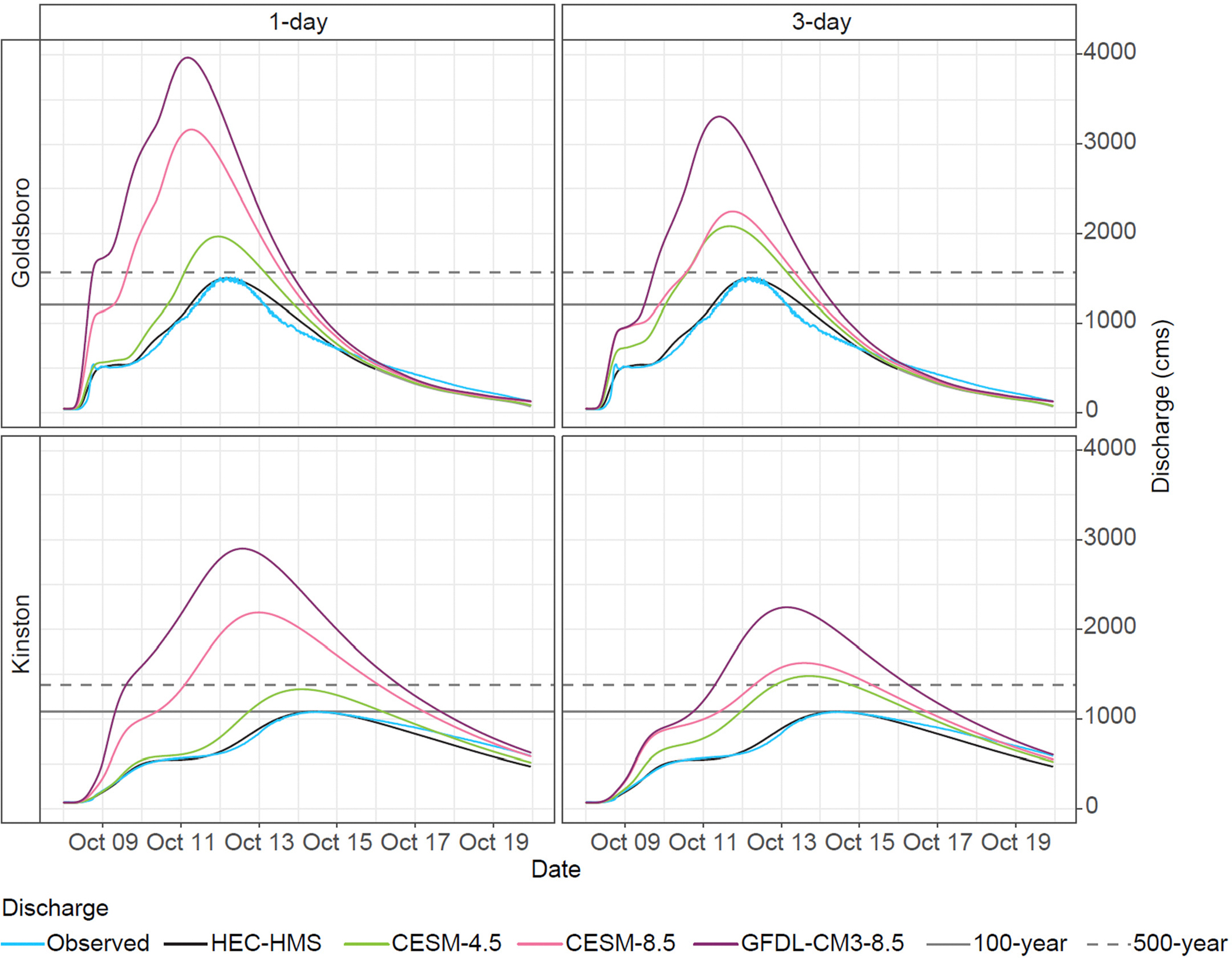
Observed and future hydrographs for the Neuse River at Goldsboro and Kinston. Horizontal lines indicate 100-year discharges (1227 cms for Goldsboro and 1076 cms for Kinston; solid gray lines) and 500-year discharges (1572 cms for Goldsboro and 1362 cms for Kinston; dashed gray lines) [26].

The observed peak discharge for the Neuse River at Goldsboro from Matthew was 1512 cms, approaching a 500-year discharge (1572 cms), and 1082 cms at Kinston, about 80% of the 500-year discharge (1362 cms) [26]. In the CESM-4.5 scenario, the 1-day rainfall Δ was 6–23%, and the 3-day rainfall Δ was −15–46% (Figure 3). The projected discharge at the end of the century is ~1.3 times that observed at Goldsboro: 1972 cms for 1-day duration and 2087 cms for 3-day duration (Figure 4), exceeding the 500-year discharge for both durations. The projected discharge at Kinston approaches a 500-year mark for 1-day duration (1335 cms) and exceeds it at 3-day duration (1482 cms; Figure 4).

Both EDDE RCP8.5 scenarios project much larger increases in discharge than RCP4.5. Under CESM-8.5, the change in 1-day rainfall ranged from −16 to 89%, and the 3-day rainfall increased by 3–49% (Figure 3). At Goldsboro, CESM-8.5 produced a peak discharge that was double the 500-year discharge (3164 cms) for the 1-day rainfall and 1.4 times the 500-year discharge (2252 cms). At Kinston, the increases were 1.6 and 1.2 times the 500-year discharge (2190 cms and 1629 cms) for 1-day and 3-day rainfall, respectively (Figure 4). Consistent with the largest rainfall Δ, the largest projected increases in discharge are associated with the GFDL-CM3–8.5 scenario, where the 1-day rainfall Δ increased by 39–112%, and the 3-day rainfall increased by 33–82% (Figure 3). The resultant water volumes at Goldsboro were 2.5 times the 500-year discharge (3965 cms) for the 1-day rainfall and 2.1 times the 500-year discharge (3310 cms; Figure 4). At Kinston, the projected peak discharges were 2.1 and 1.7 times the 500-year discharges for the 1- and 3-day events, respectively (2906 cms and 2248 cms; Figure 4).

**Figure 5.**
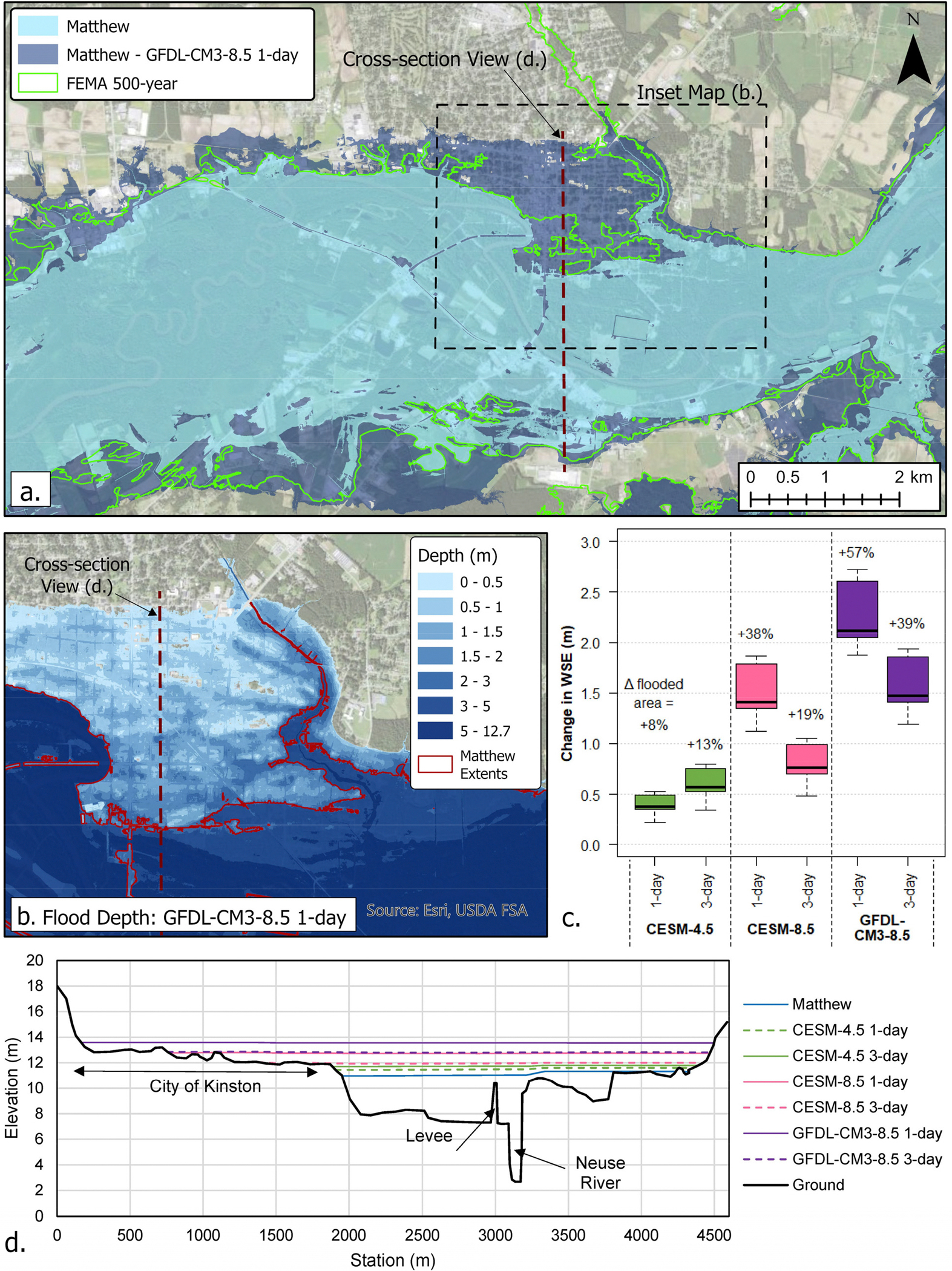
(**a**) HEC-RAS simulated change in flooding extent for the NRB at Kinston for GFDL-CM3–8.5 1-day rainfall. (**b**) Δ in the depth of flooding in the downtown Kinston area for GFDL-CM3–8.5 1-day rainfall. (**c**) Increase in WSE and percent Δ in the area flooded associated with each EDDE climate change scenario for Hurricane Matthew in the Kinston area. (**d**) Cross-section view of Δ in WSE in the Kinston area. The deltas reflect the change in period from 2025–2054 to 2070–2100.

**Figure 6.**
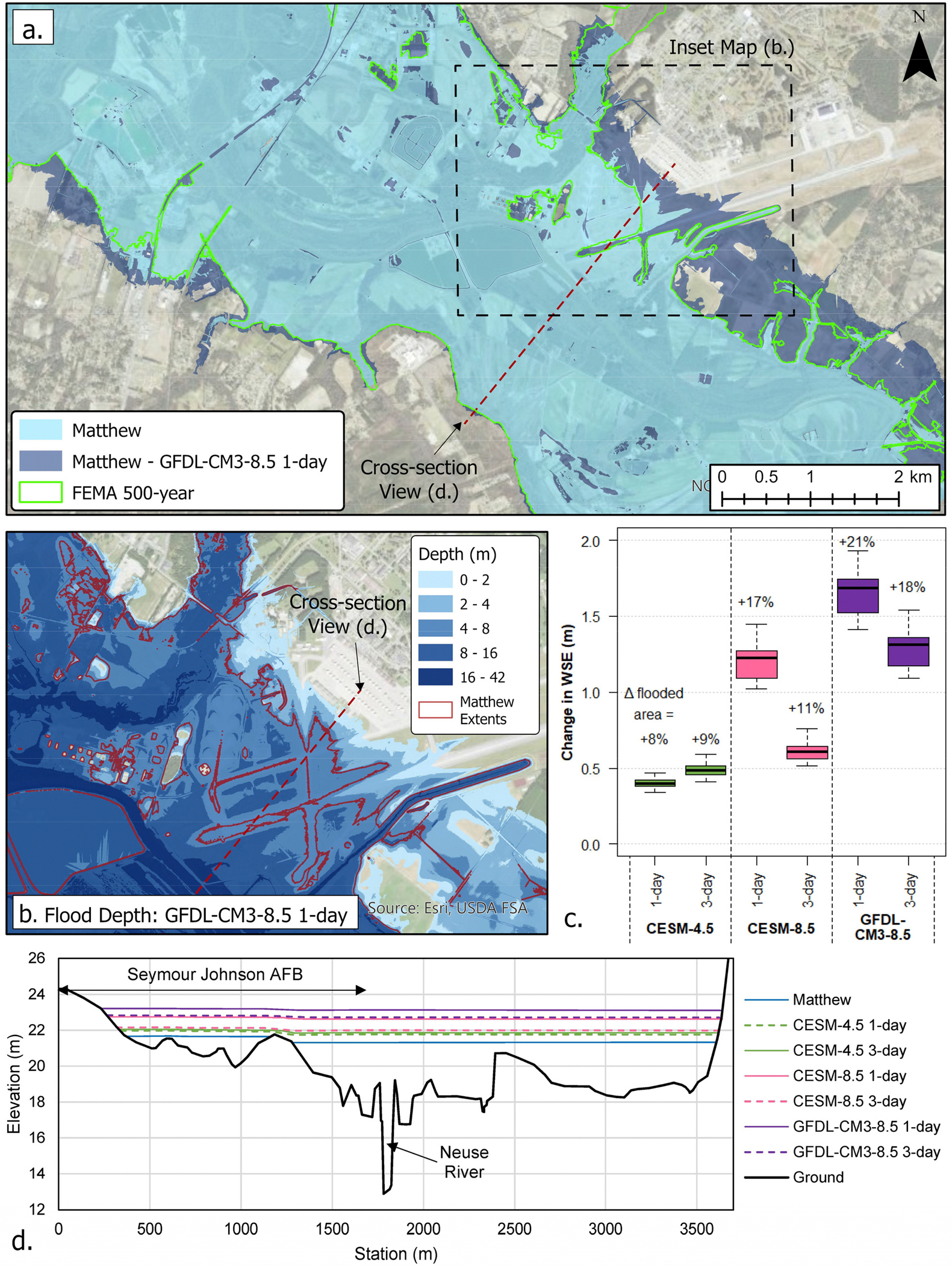
(**a**) HEC-RAS simulated change in flooding extent for the NRB at Goldsboro for GFDL-CM3–8.5 1-day rainfall. (**b**) Δ in the depth of flooding in the downtown Goldsboro area for GFDL-CM3–8.5 1-day rainfall. (**c**) Increase in WSE and percent Δ in the area flooded associated with each EDDE climate change scenario for Hurricane Matthew in the Goldsboro area. (**d**) Cross-section view of Δ in WSE in the Goldsboro area. The deltas reflect the change in period from 2025–2054 to 2070–2100.

Projected peak discharges were further used in two-dimensional HEC-RAS [25,66] models to estimate the resulting changes in the WSE and the flooding extent relative to those observed during Matthew. Here, the changes under the scenarios represent Δ between 2070–2100 and 2025–2054. For the CESM-4.5 scenario, the projected discharge resulted in 0.3–0.75 m increases in WSE in the Kinston area and 8–13% increases in inundated area (Figure 5). For Goldsboro, the projected increases in WSE under the CESM-4.5 scenario were 0.4–0.6 m and an 8–9% increase in the flooded area (Figure 6). For both RCP8.5 scenarios, WSE in the Kinston area increased by 0.5–2.5 m, leading to 19–57% increases in inundated area (Figure 5). For Goldsboro, the increases in WSE were 0.5–1.9 m, and the flooded area increased by 11–21% (Figure 6). Projected flood extents in Kinston under CESM-4.5 were comparable to the current 500-year floodplain [44], while RCP8.5 scenarios far exceeded the 500-year areal bounds (Figure 5).

## 4. Discussion

This study analyzed historical changes in rainfall characteristics in the NRB in ENC, which is a generally flat and low-lying region that is frequently impacted by tropical cyclones. This study demonstrated that changes in the most extreme observed rainfall events (99.9th percentile; Figure 1b; Table S1) strongly reflect the historical period length and the events that were included in that record. The influence of the length of the data record is also shown in recent National Climate Assessments. In the Third National Climate Assessment [74], the observed top 1% 1-day rainfall in the Northeast increased by 71% over 1958–2012. In the Fourth National Climate Assessment [75], the same region and metric increased by 55% from 1958 to 2016 and by 38% from 1901 to 2016. The same time-period dependency principle would apply to future datasets and how inclusive or selective one would be with chosen time periods. Changes in observation density may also contribute to differences in those calculations.

The issues resulting from the density of station data are analogous to the grid spacing of the modeled data. Decreasing DD grid spacing has been found in multiple studies [76] to improve the simulation of rainfall extremes. In SD, accurately simulating rainfall extremes is driven by observation data quality [40]. To minimize the impact of the horizontal resolution and inherited biases from the GCMs [71,77–79], this study focused on analyzing relative changes in future rainfall. The higher variation in Δ from SD-LOCA and SD-MACA relative to DD could be attributed to drawing from substantially more data points within the NRB: 264 (SD-LOCA) and 541 (SD-MACA) vs. 6 (DD-EDDE) and 12 (DD-CDX) (Figure S2). The different constructed analog methodologies in SD-LOCA and SD-MACA may also contribute to a larger Δ. The SD-LOCA used localized clusters to develop daily analogs, while SD-MACA used analogs developed for the CONUS, resulting in a patchwork of analogs in SD-LOCA [61,62]. The NRB subset in this study may be derived from two or more SD-LOCA clusters, which may explain the inflated variability of SD-LOCA compared to similar methods, like SD-MACA. Additional grid cells may better represent regional heterogeneity; however, the large variation in SD data also reflects stationary biases acquired from the observation history used to develop SD models [80].

The physics-based DD realizations simulate complex rainfall processes at regional scales but are computationally intensive; they typically have coarser spatial resolution and finer temporal resolution than SD. SD applies statistical relationships between local climate variables from observations and large-scale predictors, is computationally inexpensive, and has finer spatial resolution. However, SD does not consider physical processes and interactions contributing to rainfall extremes. The uncertainty and biases in large-scale processes in GCMs affect extreme rainfall events and are often inherited in downscaled datasets. Figure 2a,b show significant differences between DD and SD representations of rainfall extremes from each GCM. The stationarity bias in SD data is both temporal (by imposing historical patterns into the future and applying bias corrections) and spatial (by forcing historical changes to grid cells corresponding to weather stations) [81]. SD biases converge future realizations toward observed distributions, given the similarities between SD-LOCA and SD-MACA (e.g., Figure 2b). Nevertheless, SD datasets are attractive and ubiquitous for regional climate change impact assessments by offering numerous scenarios (e.g., the SD-LOCA ensemble includes 32 models, and only 5 are used here; Table 1). However, the scenario ensembles are often used as a weighted average, obscuring the variability of modeled extremes through averaging. DD offers a smaller ensemble (owing to its computational requirements) but can more adequately simulate intensities of extreme events [82], offering a potential amplification of extreme rainfall values, if supported by changes to the physical atmosphere. Future increases based on SD models reported in the literature are generally suppressed relative to DD, vary by GCM and durations, and are affected by the horizontal grid spacing of the downscaled data [30,81].

Observations (Figure 1 and Table S1) indicate that Kinston rainfall extremes predominantly result from TCs, while fewer than half of the rainfall extremes at Raleigh (~130 km farther inland than Kinston) are associated with TCs. The thermodynamic principles in the Clausius–Clapeyron (C-C) relationship predict a ~7% increase in the saturation water vapor pressure per 1 °C increase in temperature [83]. However, one observation- and model-based study concluded that changes over 1950–2010 were 3.0 ± 1.6% °C^−1^ and 4.3 ± 2.0% °C^−1^, respectively [84], which is more conservative than the theoretical C-C relationship. Another, observation-based study emphasized sensitivity to the rainfall frequency and presence of convection showed that the C-C relationship may be as high as 13.0% °C^−1^ [20,85,86]. Furthermore, convective available potential energy, a main driver of convective storms, is projected to increase and compensate for the decreased wind shear, which may intensify convective events [87] and their further intensification by increases in convective inhibition [88]. TCs would be altered by changes in atmospheric dynamics (i.e., Walker circulation). While the intensity of TCs may increase [89], weakening of the Walker circulation may decrease the frequency of their occurrence [90].

Relationships in the analysis of the observational data are reflected in DRA analysis (Figure 2), which enables exploring how future changes in extreme rainfall may impact river discharge and flooding in ENC using dynamically downscaled projections with discharge simulated using hydrologic and hydraulic models. DRA uses relative changes between two future periods to project future storm intensities, circumventing the use of bias correction. Although this method is demonstrated for stakeholders, some limitations and sources of uncertainty are noted. The physical forcing mechanisms for extreme rainfall were not distinguished in DRA, but follow-on studies could include more granularity. Furthermore, projections—whether directly from GCM or downscaled by any method—are unequally skilled at representing the spectrum of processes resulting in extreme rainfall [77–79,91] (e.g., DD can reflect the presence of TCs, but frequency and intensity of TCs are often underestimated). Thus, the composition of the future rainfall data used in DRA will depend on the representation of these events in GCMs and their downscaled realizations. Overall, convective events result from atmospheric processes that are not resolved by GCMs [92]. TCs in downscaled models are sensitive to spatial resolution and biases from the GCM [91]. Regardless of the limitations, the DD-CDX models produced TCs with slightly decreased storm counts and associated rainfall over the Gulf of America and Caribbean [91,93] and increased rainfall near the mid-Atlantic [91]. Although GCMs introduce uncertainties [38] and the grid spacing used for DD here is modest, follow-on studies with DD could be constructed to assess the evolution of future changes by the event type and its influence on the DRA-TC estimation. However, SD cannot be used because event types are unclassifiable due to the limitations of those datasets.

Using DD-EDDE rainfall projections and DRA, relative increases in rainfall for a design storm, Matthew, were input into the hydrologic model (HEC-HMS). The potential changes in discharge and WSE were quantified for Matthew under atmospheric conditions projected for the end of the 21st century. The DD-EDDE models used in hydrologic modeling consistently produced the highest Δ values, and it could be argued that they represent a “worst-case scenario”. However, if the sensitivity of Δ to the data sampling period is considered with the changes in future thresholds and compared to changes in historical thresholds from two different periods (Figures 1b and 2b,c), the notably highest future Δ produced by GFDL-CM3–8.5 is within the range of observed changes (Figure 2c). That is, although the relative magnitudes of the projected changes may seem dramatic, they are consistent with ongoing changes observed outside the study area. Similarly, a previous study concluded that such an extreme relative magnitude of modeled changes in rainfall for ENC is reasonable and that the magnitude of single-event rainfall has already been observed elsewhere in the southeastern U.S. [30]. Although the projected increases in discharge modeled using GFDL-CM3–8.5 may seem excessive, the modeled flow only reflects the given pluvial conditions in NRB (Figures 3 and 4) that consequently were generated based on Δ values that are within the range of previously observed changes (Figure 2). It is also consistent with hydrologic processes in a watershed, where the rainfall accumulation reaches a point at which soil infiltration and surface storage are overwhelmed, resulting in 100% of the rainfall contributing to runoff/discharge and dramatically increasing the peak discharge and flooding extent. The projected increases of 0.4–3 m in WSE and 8–57% in flooded areas above Matthew levels underscore that both future scenarios would cause catastrophic flooding and significantly increase the area susceptible to frequent flooding in ENC (Figures 5 and 6). Accordingly, Kinston is a vulnerable, low-lying community that could benefit from DRA (with appropriate caveats) to develop resilience and adaptation strategies.

Another limitation is that DRA was applied using the RPs from the most current Atlas 14 (Figure 1b) [43], which excludes almost a dozen TCs affecting ENC (such as Matthew) since its publication in 2006. Including the additional decade of data would likely reduce RPs and their confidence intervals (Cis), as well as the corresponding Δ (Tables S3 and S4). Additionally, DRA uses 30 years for each future data series to generate lengthier and extreme RPs, which generates larger CIs and may underestimate calculated intensities [94]. Yet, the Δ used in DRA originated from the mean PIDF curves, excluding the potential minima and maxima indicated by the CIs. Notwithstanding, the projected changes in future extreme rainfall, discharge, and flooding—intensified by relatively flat topography—could be catastrophic for ENC. Both CESM-8.5 and GFDL-CM3–8.5 project only small or negative Δ in rainfall intensities for RPs ≥50 years in western NRB (farther inland), juxtaposed with dramatic increases in eastern NRB. Additionally, projected increases in WSE and flooding extent in Kinston were greater than in Goldsboro due to the topography of the Neuse River valley. Lower longitudinal slope in Kinston (0.0002 vs. 0.0003 m m^−1^ in Goldsboro) and wide Kinston floodplains that drastically narrow downstream of the town (Figures 5 and S4) would favor flooding. Furthermore, the changes in WSE and flooding extent were lower for Goldsboro because the flood extents of Matthew had already reached the Goldsboro area alluvial relic terraces on the left bank of the river, and the Neuse River valley wall on the right bank (Figure S4). Hence, the gradual change in topography combined with larger increases in extreme TC rainfall in coastal areas could be especially problematic for ENC.

Increased atmospheric warming is correlated with an increased risk of flooding [10]. However, overwhelming evidence of intensifying rainfall extremes has not yet been associated with increases in flooding frequency and extent in historical data [17,94,95] due to complex natural (e.g., reductions in soil moisture) [96] and anthropogenic hydrologic processes (e.g., flood management) that could mitigate future floods. For example, increases in water usage in the growing Raleigh metropolitan area (at the head of the Neuse River Basin), increased evaporation from reservoirs, and flood management actions may alleviate the dramatic potential consequences of floods in the future. These results may motivate stormwater managers and stakeholders to plan for the possibility of TCs producing rainfall and flooding beyond the current 500-year floodplain in areas already vulnerable to frequent flooding, such as Kinston. These areas would be prone to even more devastating impacts, threatening populated areas not yet directly impacted by recent observed TCs. The results also emphasize that future datasets should be approached with caution; i.e., users must examine downscaling methods, evaluate model performance, and consider the influence of the length of the timeseries used. The projected increases in extreme rainfall and resulting flooding would be exacerbated in coastal areas—many of which have regionally high rates of poverty—by other factors (e.g., sea-level rise, intensifying TC wind speeds, and storm surge) that were not included in this study.

It is important to emphasize that this study focused on testing the application of future climate data in hydrologic and hydraulic modeling and did not address the inclusion of data reflecting future changes in population density, land use, or sea-level rise. The HEC-HMS model used here, developed by NCDOT NCEM ([26], Supplementary Material), captures the hydrology of a specific event in a particular landscape, and future scenarios reflect changes in future rainfall only. However, NCDOT NCEM [26], in coordination with other agencies and stakeholders, also developed twelve strategies for flood mitigation in the NRB basin using different population and water management strategies. The presented concept of using DRA in hydrologic and hydraulic modeling is applicable in other watersheds and can provide more insights into developing mitigation and resilience scenarios to better prepare for intensifying rainfall events.

## Supplementary Material

SupplementaryMaterial

**Supplementary Materials:** The following supporting information can be downloaded at: https://www.mdpi.com/article/10.3390/w17152228/s1, Figure S1: Peak streamflow in Goldsboro and Kinston; Figure S2: Grid spacing over the Neuse River Basin from the downscaled datasets used in this study; Figure S3: 2nd-quartile storm used for Matthew in HEC-HMS; Figure S4: Topography and flood extents from the calibrated and future Matthew; Table S1: Observed daily rainfall events above 99.9th percentile; Table S2: Weather Research and Forecasting (WRF) model configurations for datasets in the study; Table S3: Change in precipitation probability statistics applied in the study, by sub-basin; Table S4: Change in precipitation probability statistics applied in the study, by return period. Table S5: Sample of parameter calculations and adjustments. From NCDOT NCEM report.

## Figures and Tables

**Table 1.** Members of the CMIP5 ensemble [38] that were downscaled and used in this study. The first three models were downscaled by the U.S. EPA (“DD-EDDE”)and the last three within the North American Coordinated Regional Downscaling Experiment (“DD-CDX”). Each dynamically downscaled dataset was produced with the Weather Research and Forecasting (WRF) model, and the results were not bias corrected. Descriptions of the WRF model configurations are in Table S2. Where possible, each of the dynamically downscaled datasets was paired with statistically downscaled data from the same GCMs from the Localized Constructed Analogs (“SD-LOCA”) and the Multivariate Adaptive Constructed Analogs “SD-MACA” (MACAv2-METDATA) ensembles.

Name	GCM	RCP	DD Ensemble and Horizontal Grid Spacing	Used in HEC-HMS	Years	SD Equivalent Source and Grid Spacing
“CESM-4.5”	Community Earth System Model version 1 (a.k.a., the fourth version of the Community Climate System Model–CCSM4) [32]	4.5	DD-EDDE 36 km	Yes	1975–2005 2025–2099	NA
“CESM-8.5”		8.5		Yes		MACA 4 km LOCA 7 km
“GFDL-CM3-8.5”	Geophysical Fluid Dynamics Laboratory Coupled Model [35]	8.5	DD-EDDE 36 km	Yes	1995–2005 2025–2099	LOCA 7 km
“HAD-GEM2-ES-8.5”	Hadley Centre Global Environment Model version 2 [33]	8.5	DD-CDX 25 km	No	1950–2005 2006–2099	MACA 4 km LOCA 7 km
“MPI-ESM-MR-8.5”	Max Planck Institute Earth System Model mixed resolution [34]	8.5		No		LOCA 7 km
“GFDL-ESM2M-8.5”	GFDL Earth System Model [36,37]	8.5		No		MACA 4 km LOCA 7 km

## Data Availability

Data used in this analysis are available via the U.S. EPA’s Environmental Dataset Gateway (http://edg.epa.gov [accessed on 24 July 2025]). Subsets of the EDDE dataset are available at https://registry.opendata.aws/epa-edde-v1/ (referenced by https://doi.org/10.23719/1530964 [accessed on 24 July 2025]).

## Abbreviations

The following abbreviations are used in this manuscript:

U.S.: United States
CONUS: Contiguous U.S
NC: North Carolina
ENC: Eastern NC
EPA U.S.: Environmental Protection Agency
USGS U.S.: Geological Survey
NOAA: National Oceanic and Atmospheric Administration
FRIS: Flood Risk Information System
FEMA: Federal Emergency Management Agency
NCDOT: NC Department of Transportation
NCEM: NC Division of Emergency Management
Raleigh State Univ: Weather station located at NC State University in Raleigh, NC, part of the NC State Climate Office’s ECONet.
Raleigh AP: Primary (first order) weather station located at the Raleigh-Durham Airport in Morrisville, NC. The AP stands for airport.
Kinston 7SE: Weather station located south-east from Kinston.
Kinston AG: Weather station located at Cunningham Research Station, an agricultural
Research, NC: research station for N.C. State University.
TCs: Tropical Cyclones
TCFF: Freshwater flooding induced by extreme rainfall associated with TCs.
Matthew: Hurricane Matthew
Matthew 2100: Future realizations of Hurricane Matthew by year 2100.
p1, p2: Period one, period two.
Delta (Δ) Change: Difference between p1 and p2.
PIDF: Precipitation intensity–duration–frequency
DRA: Design rainfall approach
RP: Return period
AMS: Annual maximum series
L-moments: Statistics used to summarize the shape of a probability distribution.
GEV: Generalized extreme value distribution
RFA: Regional frequency analysis
NSE: Nash–Sutcliffe model efficiency coefficient
R^2^: Coefficient of determination
WSEs: Water surface elevations
C-C relationship: Clausius–Clapeyron relationship
HEC-HMS: Hydrologic Engineering Center–Hydrologic Modeling System
HEC-RAS: Hydrologic Engineering Center–River Analysis System
WRF: Weather Research and Forecasting
DD: Dynamically downscaled
SD: Statistically downscaled
GCMs: Global climate models
RCMs: Regional climate models
CMIP5/CMIP6: Coupled Model Intercomparison Project Phase 5/Phase 6
RCPs (RCP4.5, RCP8.5): Representative Concentration Pathways (scenarios) expressed as changes in radiative forcing values (here, 4.5 and 8.5 W/m^2^, respectively) between 1750 and 2100.
DD-EDDE: DD data from EPA Dynamically Downscaled Ensemble
DD-CDX: DD data from NA CORDEX North American Coordinated Regional Downscaling Experiment
SD-MACA: SD from Multivariate Adaptive Constructed Analogs (MACAv2-METDATA
SD-LOCA: Localized Constructed Analogs
CESM-4.5/-8.5: GCM from Community Earth System Model (CESM) version 1 (a.k.a., Community Climate System Model v4– CCSM4) under RCP4.5 and RCP 8.5 scenarios
GFDL-CM3–8.5: GCM from Geophysical Fluid Dynamics Laboratory Coupled Model v3 under RCP 8.5 scenario.
HAD-GEM2-ES-8.5: GCM from Hadley Centre Global Environment Model v 2 under RCP 8.5 scenario.
MPI-ESM-MR-8.5: GCM from Max Planck Institute Earth System Model mixed resolution under RCP 8.5 scenario.
GFDL-ESM2M-8.5: GCM from Geophysical Fluid Dynamics Laboratory Earth System Model under RCP 8.5 scenario.
