# Supplementary material for: Assessing Flooding from Changes in Extreme Rainfall: Using the Design Rainfall Approach in Hydrologic Modeling
Source: Water (Basel). Author manuscript; Available in PMC 2026 May 5. (PMC13137454; doi:10.3390/w17152228)
Supplement: SupplementaryMaterial [file NIHMS2152887-supplement-SupplementaryMaterial.pdf]

1. Observational Data

Figure S1

Peak streamflow in Goldsboro (USGS-02089000) and in Kinston (USGS-02089500) for 1928–2020. The completion of the Falls Lake Reservoir in 1981 is marked. Data were obtained from https://nwis.waterdata.usgs.gov/nwis/peak?site_no=02089000&agency_cd=USGS&format=rdb for Goldsboro and from https://nwis.waterdata.usgs.gov/nwis/peak?site_no=02089500&agency_cd=USGS&format=rdb for Kinston.

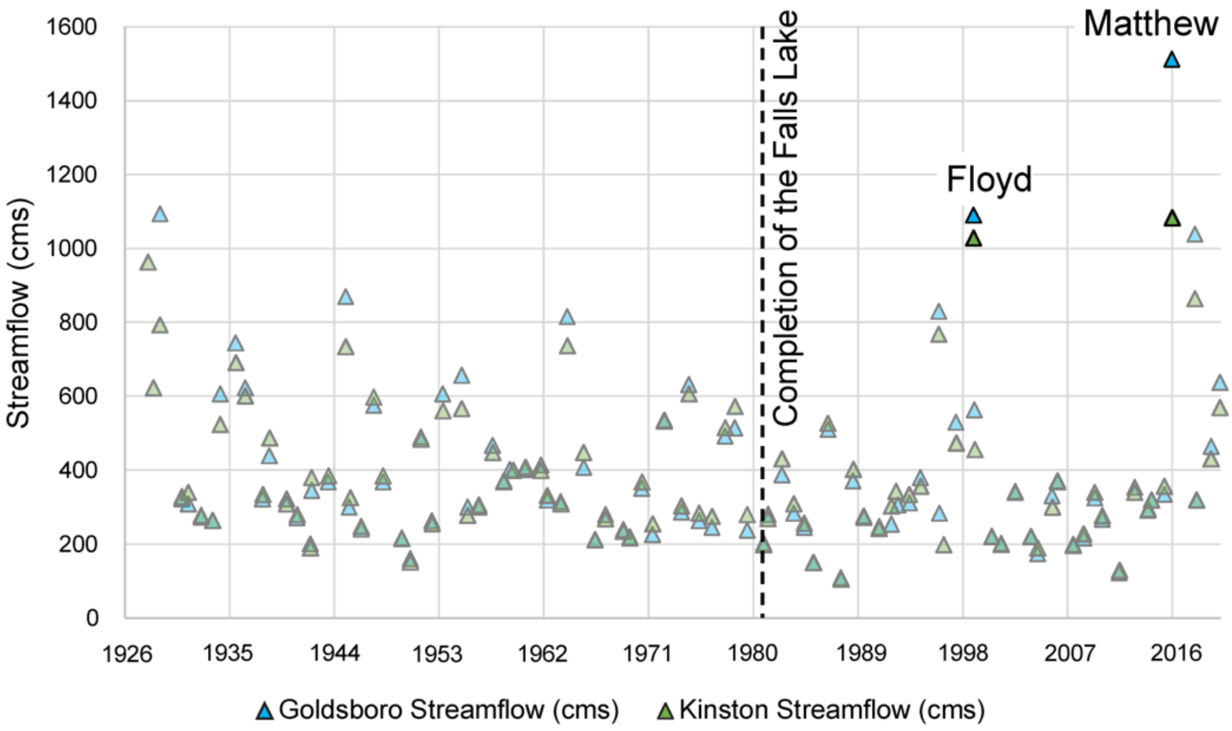

Table S1

Observed daily rainfall events above 99.9th percentile. If the event is associated with a tropical cyclone (TC), the storm name is provided, if applicable.

Raleigh (1887–2021)				Kinston (1900–2018)			
	Date	Rainfall (mm)	Event type		Date	Rainfall (mm)	Event type
1	1908-08-24	145	*No TC*	1	1908-07-31	152	TC
2	1915-08-03	134	*No TC*	2	1942-07-24	118	*No TC*
3	1929-10-01	151	*No TC*	3	1946-07-16	123	TC
4	1931-07-04	123	*No TC*	4	1955-08-13	199	Connie
5	1940-08-14	111	TC	5	1960-09-12	173	Donna
6	1957-05-11	110	*No TC*	6	1969-11-02	122	*No TC*
7	1963-11-06	116	*No TC*	7	1985-08-19	140	Danny
8	1986-08-20	106	*No TC*	8	1991-04-20	116	*No TC*
9	1996-09-06	126	Fran	9	1996-07-13	116	Bertha
10	1997-07-24	106	Danny	10	1996-09-06	145	Fran
11	1999-09-05	123	Dennis	11	1998-08-27	136	Bonnie
12	2002-10-11	136	Kyle	12	1999-09-05	140	Dennis
13	2006-06-14	143	Alberto	13	1999-09-16	300	Floyd
14	2008-09-06	120	Hanna	14	2005-10-08	126	Tammy
15	2011-08-06	110	*No TC*	15	2006-09-01	244	Ernesto
16	2013-06-07	131	*No TC*	16	2010-09-28	128	*No TC*
17	2014-07-15	107	*No TC*	17	2010-09-30	123	*No TC*
**18**	**2016-10-08**	**164**	**Matthew**	18	2011-08-27	243	Irene
19	2017-04-24	115	*No TC*	19	2015-05-11	122	Ana
#	2021-10-09	126	*No TC*	20	2016-09-03	115	Hermine
				**21**	**2016-10-09**	**215**	**Matthew**
				22	2017-04-25	118	*No TC*
				23	2018-09-14	158	Florence
				24	2018-09-15	141	Florence

2. Modeled Data

Figure S2

Grid spacing over the Neuse River Basin from the downscaled datasets used in this study. DD-EDDE [1,2] (36-km; black; 6 grid cells), DD-CDX [3] (25-km; yellow; 12 grid cells), SD-LOCA [4] (7-km; pink; 264 grid cells) and SD-MACA [5,6] (4-km; navy blue; 541 grid cells).

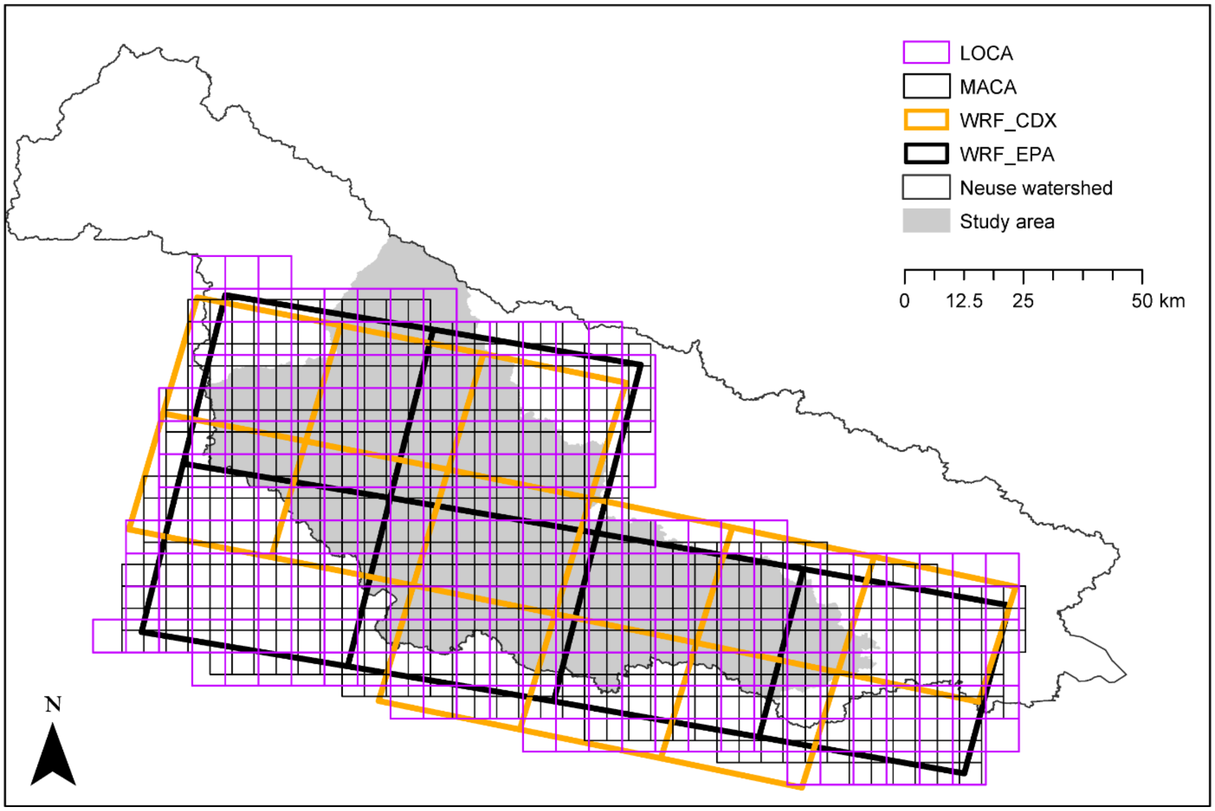

Table S2

Weather Research and Forecasting (WRF) model [7] configurations used by the U.S. EPA [1,2] ('CESM-4.5', 'CESM-8.5', 'GFDL-CM3-8.5') and as part of the North American Coordinated Regional Downscaling Experiment (NA-CORDEX) [3] ('HAD-GEM2-ES-8.5', 'MPI-ESM-MR-8.5', 'GFDL-ESM2M-8.5').

	GFDL-CM3	CESM-CCSM4	HAD-GEM2-ES	MPI-ESM-MR	GFDL-ESM2M
**GCM source**	NOAA	NCAR	MOHC	MPIM	NOAA
**GCM Resolution**	2° × 2.5°	0.875° × 1.25°	1.25° × 1.875°	1.8653° × 1.875°	2. 5° × 2.5°
**Scenarios**	RCP8.5	RCP4.5 and RCP8.5	RCP8.5	RCP8.5	RCP8.5
**Downscaling Institution**	UNC and U.S. EPA	U.S. EPA	NCAR	UA	NCAR
**WRF Version**	v3.6	v3.4.1	v3.5.1		
**WRF Domain**	108-36-km, two-way nest	36-km	25-km		
**Historical period**	1995–2005	1975–2005	1950–2005		
**Future period**	2025–2100		2006–2099		
**Bias correction**	none				
**Dynamics**	Non-hydrostatic, Compressible				
**Land-Surface Model**	NOAH				
**LSM Input Databases**	USGS 24-category data				
**Vegetation Types**	24 types				
**Microphysics**	WSM6		WSM3		
**CPS**	Kain-Fritsch w/ radiative feedback, Herwehe et al. (2014) [8]		Kain-Fritsch		
**Vertical Coordinate**	Terrain-following hydrostatic pressure coordinate, 35 levels		Terrain-following hydrostatic pressure coordinate, 28 levels		
**Spectral Nudging**	Spectral nudging above PBL, Otte et al. (2012) [9]		Spectral nudging at all levels and for wavelengths greater than ~1000 km		
**Spin-up Period**	1 month (GFDL) and 3 months (CESM)		1 to 2.5 years depending on simulation		
**Length of Timestep**	180 s		150 s		
**Daily T_{max} and T_{min}**	hourly		3-hourly		
**Longwave/ Shortwave Radiation**	RRTMG/ RRTMG		RRTM/ Goddard		
**PBL model**	YSU		MYJ		
**Driving Ocean BC var**	tos, sic		tos, no sic	tos, sic	
**Sea Ice Characteristics**	sea ice provided as lower boundary condition		threshold set to 272.0 K; fractional sea ice if sic = LBC	sea ice provided as lower boundary condition	
**Lake Temperatures**	FLake, Mallard et al. (2014) [10]	Community Land Model, Spero et al. (2016) [11]	Default WRF interpolation from nearby ocean SSTs		
**Uniform Aerosols**	yes	NA in WRF v3.4.1	yes		
**Radiative Forcing**	Follows RCPs	Standard- other not available in WRF version used			

**Acronyms: National Oceanic and Atmospheric Administration (NOAA), National Center for Atmospheric Research (NCAR), Max Planck Institute for Meteorology (MPIM), Met Office Hadley Centre (MOHC), University of North Carolina (UNC), United States Environmental Protection Agency (U.S. EPA), University of Arizona (UA), Convective Parameterization Scheme (CPS), Planetary Boundary Layer (PBL).*

3. HEC-HMS and HEC-RAS Models

Excerpts from NCDOT NCEM report [12]. Unless annotated with letter S the references to figures and tables in this excerpt correspond to the figures and tables in the NCDOT NCEM report [12].

3.1 Datasets

- **Rainfall** – Average annual rainfall in the Neuse River Basin ranges from 44.7 inches to 56.9 inches with the larger totals occurring in the eastern portion of the basin. To characterize a flooding event, the point frequency rainfall depth is used. Estimates for these values for different locations within the Neuse River Basin can be acquired from the National Ocean and Atmospheric Administration (NOAA) Atlas 14 Volume 2 (Bonnin et al., **2006)** or digitally from NOAA's Precipitation Frequency Data Server at https://hdsc.nws.noaa.gov/hdsc/pfds/. In the full report these statistics are available for time periods ranging from 5 minutes to 60 days. The temporal distribution of rainfall for a storm even can have an impact on the flooding response. A storm with a steady rain for its duration will result in a different flooding response than a storm where the majority of the rainfall is concentrated into a small portion of the overall length of the storm. Figure S3 shows a temporal distribution for a second quartile 24-hour duration storm. This figure is adopted from Atlas 14 Volume 2 (Bonnin et al., **2006)**.
- **Rainfall Data –** The National Weather Service (NWS) operates a network of rainfall gages across North Carolina, the majority of which are part of the Cooperative Observer Program (COOP) network. COOP network gages in North Carolina have some of the longest periods of rainfall records in the State, including several with records in excess of 100 years. The State Climate Office of North Carolina (SCO) compiles and archives records from more than 37,000 North Carolina weather sites, including those in the COOP network, in the North Carolina Climate Retrieval and Observations Network of the Southeast (CRONOS) Database. The SCO compiled monthly rainfall records from eight long-term rainfall gages in and adjacent to the Neuse River Basin for use in this investigation.
- **Stream Gages –** The United States Geological Survey (USGS) currently maintains 24 stream gages in the Neuse River Basin. Additionally, there are 8 sites that are no longer active. Figure 4.3 in this report shows a map of the Neuse River Basin with gages that were used for calibration of the project hydrologic model. Major floods along the Neuse River occur most often in association with hurricanes or tropical storms.

3.2 Model parameterization

- **Rainfall Runoff Model –** The existing National Flood Insurance Program (NFIP) hydrologic data for the Neuse River was developed using regression analysis calibrated to discharge gage data. This is an excellent method for determining peak discharges, however, in order to fully assess mitigation options it was necessary to develop a hydrologic model that takes into account volume and timing of the flood. Regression analysis does not do this. To account for timing and volume, a high-level rainfall-runoff model was created for this effort. The USACE's HEC-HMS v4.2 software package was selected for the hydrologic calculations. For additional information on development of the hydrologic data and the data inputs please refer to Appendix G: Neuse River Draft Hydrology Report.
- **Basin Delineation –** Sub-basins within the Neuse River Basin were delineated using a 50-foot hydro-corrected grid developed from the LiDAR data collected between January and March 2001 by NCEM in support of the North Carolina Floodplain Mapping Program (NCFMP). Basins were delineated with an approximate size of 50 square miles. This is a large basin size for a hydrologic analysis but was deemed appropriate for this project level analysis. Falls Lake, Crabtree Creek upstream of HWY

US 1, and Contentnea Creek upstream of Hookerton were delineated as one basin and a discharge gage with a specified hydrograph was used in the model at these locations. The Hurricane Matthew hydrograph was used for the calibration storm. The discharge gage hydrographs were scaled for the frequency event runs based on the frequency rainfall depths in the basin versus the depths recorded during Hurricane Matthew.

- **Curve Number Development –** Curve numbers are used to describe the amount of rainfall that makes it to the stream as opposed to being intercepted by vegetation, absorbed into the soil, or otherwise prevented from contributing to riverine flooding. The Soil Conservation Service (SCS) Curve Number method was used to compute runoff depths and losses. Inputs for this method are land use and hydrologic soil group. Land use data was established based on the 2011 National Land Cover Database (NLCD) developed by the Multi-Resolution Land Characteristics Consortium. Soil type information was acquired from the Natural Resources Conservation Service (NRCS, formerly SCS). Table 4.1 in the NCDOT NCEM report [12] shows the curve number matrix used to estimate curve numbers for each basin. These values are based on antecedent moisture condition II (AMC II), which implies an average moisture condition for the soil.
- **Time of Concentration –** The SCS Unit Hydrograph was used for the hydrologic model. The default peaking factor of 484 was maintained. The peaking factor is a unitless conversion constant. The value 484 is the result of assuming that the recession limb is 1.67 time the rising limb of the hydrograph. A change to this value was considered due to the largely rural and relatively flat slope in much of the study area, but when considering the shape of the recorded hydrographs during the Hurricane Matthew event it was determined that the default parameter was acceptable. The lag time for a basin can be thought of as how long it takes from the peak of the rain event until the peak of the flooding event. Lag times were initially developed using both the velocity method and the watershed SCS lag equation. The velocity method yielded times that were unreasonably short and was therefore not selected. Lag times were developed using the SCS lag equation and were calibrated using hydrograph data recorded at gages in the basin during Hurricane Matthew. The SCS lag equation is shown below:

$$L = \frac{\ell^{0.8}(S+1)^{0.7}}{1{,}900Y^{0.5}}$$

where:
L = lag, h
T_c = time of concentration, h
ℓ = flow length, ft
Y = average watershed land slope, %
S = maximum potential retention, in
$= \frac{1{,}000}{cn'} - 10$
where:
cn' = the retardance factor

More information on the SCS lag method can be found on the NRCS website. https://policy.nrcs.usda.gov/OpenNonWebContent.aspx?content=27002.wba
Results of these calculations required an average adjustment factor of approximately +90% in order to match peak timing at gaged sites. This equation was originally developed for computation of lag times in rolling hills on basins with much smaller drainage areas so the equation was not expected to yield accurate results without calibration, but it did serve as a good starting point and help provide a consistent basis from which adjustments could be applied.

Table S5

Sample of parameter calculations and adjustments. From NCDOT NCEM report [12]

Basin	SCS Lag Eq.	*l* length (ft)	CN	AMC III	CN Adj. %	Adj. CN	Y Slope (%)	S (in)	L (hr)	Lag (min)	AF	Final lag (min)	Final lag (hr)	Tc (hr)
**Goldsboro**	**479**	**36690**	**56.5**	75.4	**15%**	**65.0**	**4.23**	**5.4**	**4.2**	**252.2**	**1.90**	479	4.20	7.0
**Kinston**	**516**	**50217**	**64.4**	81.3	**13%**	**72.8**	**3.17**	**3.7**	**5.1**	**303.8**	**1.70**	516	5.06	8.4

- **Reach Routing –** Channel routing helps take into account the time water spends travelling downstream from one basin to the next. Channel routing of the discharges was performed using the Muskingum-Cunge method. Channel and overbank roughness parameters as well as 8-point cross sections were developed based on model cross sections in the FIS hydraulic models provided by NCFMP.
- **Rainfall Depths** - Gridded rainfall data from the Hurricane Matthew event was acquired from the NCEM Resilient Redevelopment effort and used as input for the hydrologic model. A 24-hour duration storm was selected for the model. The temporal distribution was based on the Atlas 14 Volume 2 (Bonnin et al., 2006) 2nd quartile storm. This distribution was selected based on a comparison of the rainfall data from the Hurricane Matthew event to rainfall data collected at National Weather Service reporting sites for the event in Raleigh and Lumberton. Figure 4.2 in the NCDOT NCEM report [12] shows the selected storm distribution with the Matthew rainfall data from the Raleigh observation station overlaid on the distribution. The cumulative recorded rainfall data is the red line on the graph. The 50% probability from the 2nd quartile storm was used. More information on the rainfall distribution can be found in NOAA's Atlas 14 Volume 2 publication (Bonnin et al., 2006). Incremental rainfall depths based on the Atlas 14 (Bonnin et al., 2006) curves were entered into the HEC-HMS model for each basin. For more information on the rainfall data inputs Frequency discharges were developed from gridded rainfall data acquired from Atlas 14 (Bonnin et al., 2006). The gridded data was used to determine rainfall depths for each of the studied frequencies including the 10-, 4-, 2-, 1-, 0.2-, and 0.1-percent annual chance events. The rainfall depths were applied on a basin-by-basin basis. Some generalization of the depths was used for ease of input, but depths remained within 5% of the computed values. For locations using input in the form of a discharge gage based on Hurricane Mathew discharge data (Crabtree Creek and Contentnea Creek), the hydrographs were adjusted to match the frequency events by using a linear factor based on rainfall depths. For example, for the watershed upstream of Crabtree Creek at US1 the 4% annual chance rainfall depth was computed to be 5.9". This is 83% of the Matthew rainfall depth of 7.1". For the source hydrograph at this location for the 4% annual chance event, the discharges recorded at 15-minute intervals at the gage during Hurricane Matthew were multiplied by 0.83 and input into the model. Additional detail on use of discharge gages in the model can be found in Appendix G of the report.

3.3 Calibration

Hurricane Matthew was chosen as the calibration storm for the HEC-HMS model. The model was calibrated in an attempt to replicate the peak discharges, total flood volumes, and flood peak timing at each of the gaged sites in the river basin. Calibration was achieved by making adjustments to the computed basin curve numbers, lag times, and the channel routing parameters.

- **Curve numbers** Starting curve numbers were based on AMC II and were adjusted based on reported volumes at gages during the calibration storm. Hurricane Matthew occurred during a time when there were moist soil conditions, which is typical for the basin wide flooding events that are being considered as part of this study. Similar moist soil conditions existed during Hurricane Fran in 1996 and Hurricane Floyd in 1999. All adjusted curve numbers fall between AMC II and AMC III values, which means the soils were at a more than average saturation point at the start of the Hurricane Matthew rainfall event. Because of this, the computed basin curve numbers needed to be adjusted up to reflect an increased percentage of precipitation running off into waterways. These adjustments were made based on reported volumes at gages during the calibration storm. A table showing the computed curve numbers as well as the adjusted curve numbers that were used in the HEC-HMS model is provided in Appendix G of the NCDOT NCEM report [12]. All adjusted curve numbers fall between AMC II and AMC III values. In addition to using curve numbers for calibration, basin lag times and channel routing parameters were adjusted to calibrate to the peak discharge and the time of arrival of the peak at each gage location. Raw lag times developed using the SCS lag equation required an average adjustment factor of approximately +90% in order to match peak timing at gaged sites. This equation was originally developed for computation of lag times in rolling hills on basins with much smaller drainage areas so the equation was not expected to yield accurate results without calibration, but it did serve as a good starting point and help provide a consistent basis from which adjustments could be applied. Lag time computations are provided in in Appendix G of the NCDOT NCEM report [12] end a sample from two basins is given in Table S5.
- **Comparison to Flood Insurance Study (FIS) Discharges** – As noted above the hydrologic model for this project was calibrated to Hurricane Matthew. All storms have many variables that contribute to magnitude of flooding. Some of these include duration, antecedent moisture condition, intensity, direction of movement, and spatial distribution of rainfall depth. The discharges reported in community flood insurance studies are generally developed using regional regression equations based on hydrologic regions and adjusted to nearby gage records as appropriate. Some studies use rainfall runoff models calibrated to a typical storm and then verified using additional storms or regression confidence limits. Due to the difference in how FIS discharges are developed, the Matthew calibrated discharges, also referred to as the project discharges, will differ from the FIS discharges. Table 4.4 in the NCDOT NCEM report [12] shows a comparison of the FIS discharges to the project discharges at selected locations on the Neuse River. Drainage area in the table was adjusted to remove the non-contributing area upstream of Falls Lake Dam. Variances in the modeled 100-year return interval discharges versus the FIS discharges range from -30% just upstream of the confluence of Contentnea Creek to +2% at the USGS gage site at Arrington Bridge Road in Goldsboro. The modeled discharges are generally lower than discharges in the FIS models. As noted in Table 4.3 of the NCDOT NCEM report [12] peak discharges match quite well with recorded Hurricane Matthew discharges, which is not surprising since the model was calibrated to the Matthew event.

- **Hydraulic Modeling Approach** – The hydraulic model is used to calculate the water surface for a particular storm event. For this project the hydraulic models developed for the Neuse River by the NCFMP were used. In order to establish the base condition to which mitigation strategies could be compared, the hydraulic model was updated with project discharges from the calibrated HEC-HMS model for each of the 6 frequency distributions being considered and for the Hurricane Matthew discharges. Slight revisions to the channel and overbank roughness coefficients were made in order to calibrate the hydraulic model using the Matthew discharges and high-water marks collected following the flood.

Figure S3
Selected, temporal distribution for 24-h, 2nd-quartile storm for the Ohio River Basin and Surrounding States (Atlas 14) [13] in grey, with the cumulative rainfall from Matthew recorded at the Raleigh AP station in blue dots and 50% probability from the 2nd-quartile storm used for Matthew in HEC-HMS in the red, dashed line on the graph.

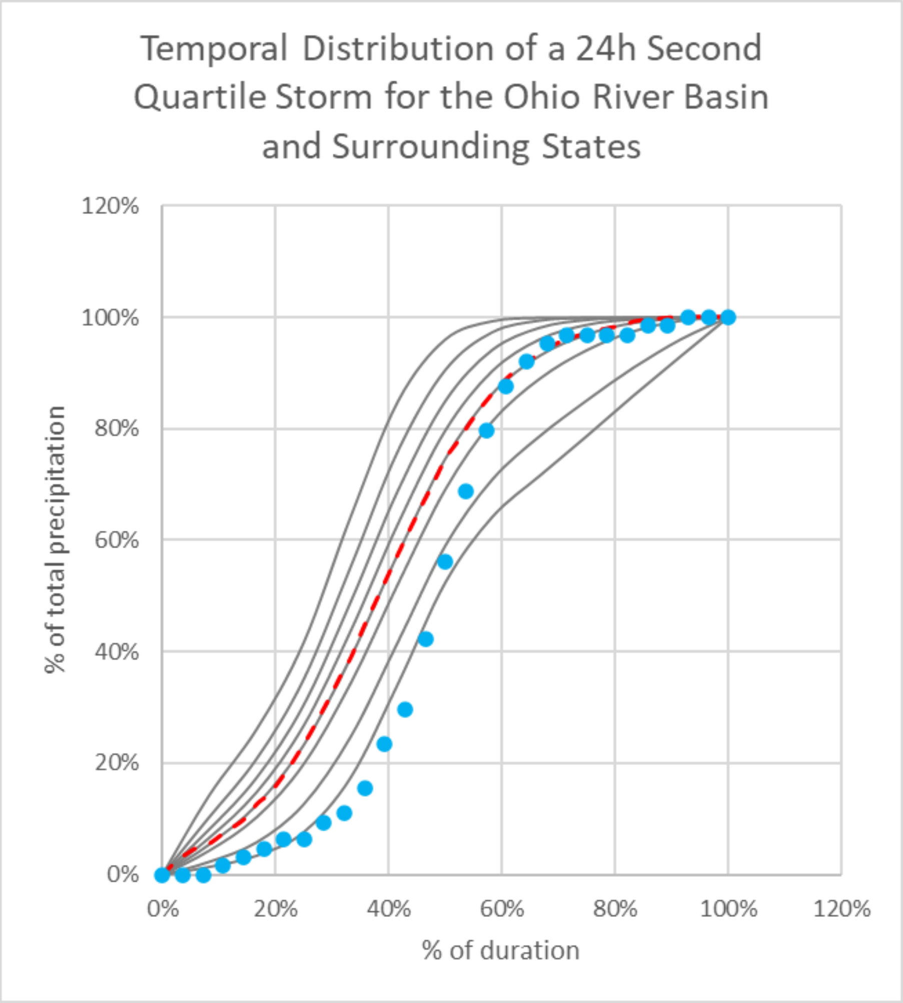

Design Rainfall Approach

Figure S4
Topography and flood extents from the calibrated Matthew (HEC-HMS) and the future GFDL-CM3-8.5 1-day scenario, which had the highest changes of the scenarios used in the study. Figure includes calibrated Matthew errors in comparison to observed high-water marks for (a) Kinston and (b) Goldsboro. For GFDL-CM3-8.5 1-day scenario the inundation extents to Neuse River relic terraces and to the walls of the Neuse River valley. In Goldsboro, the flooding during Matthew already reached the Neuse River valley walls, so the increase in flooding area for the future scenarios is less dramatic than in Kinston.

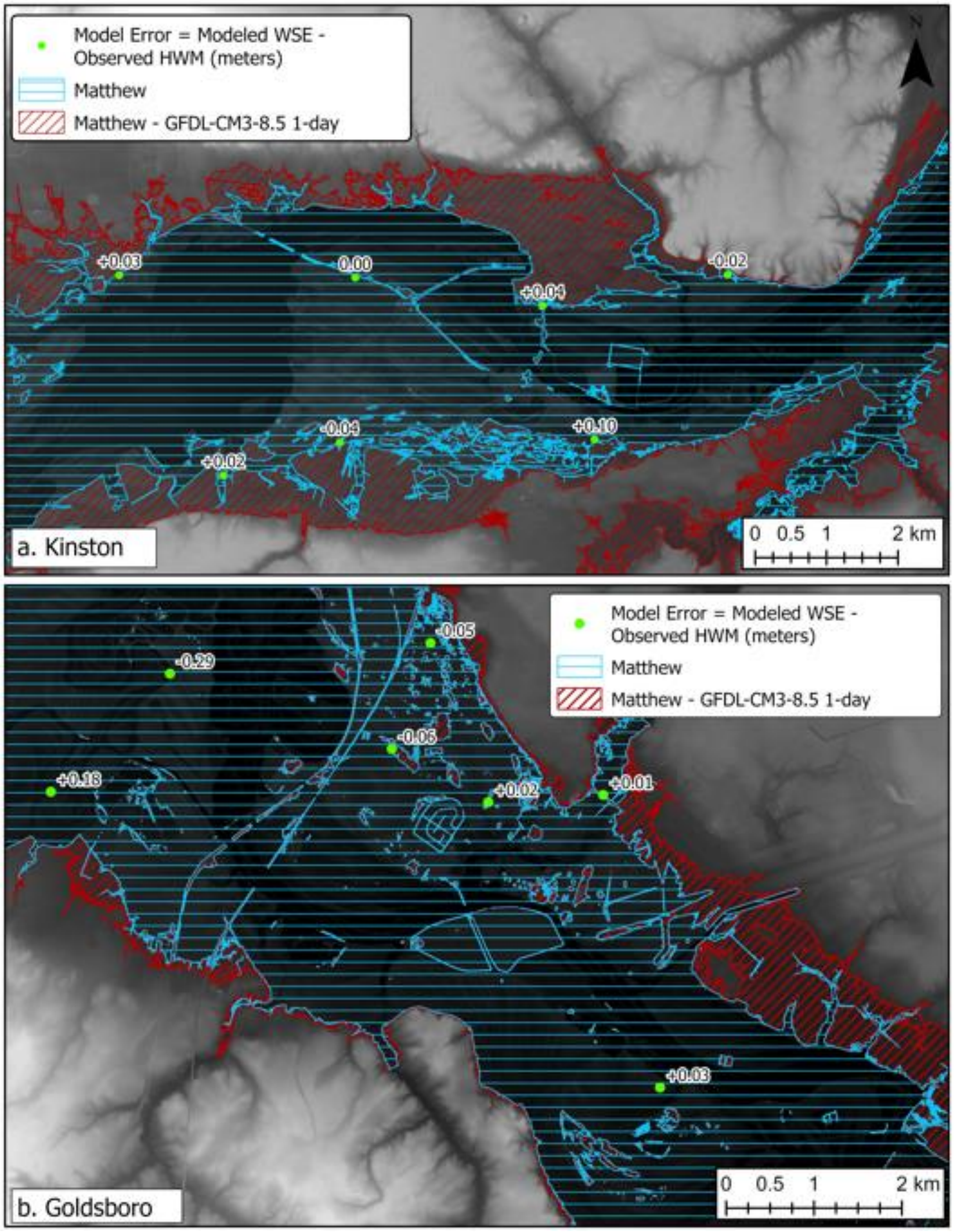

Table S3
Change (Δ) in precipitation probability statistics from 2025–2054 to 2070–2099 in the Neuse River Basin applied to design storm Matthew scenarios. DD-EDDE cell number corresponds with Jalowska et al. (2021) [14].

DD-EDDE Cell #	Watershed	Obs. Rainfall	Return Period (yr)	Δ CESM 4.5 24h	Δ CESM 8.5 24h	Δ GFDL-CM3 8.5 24h	Δ CESM 4.5 72h	Δ CESM 8.5 72h	Δ GFDL-CM3 8.5 72h
**MAX**				23%	89%	112%	46%	49%	82%
**MIN**				6%	-16%	39%	-15%	3%	33%
5	Black1	273	1000	11%	89%	76%	-15%	32%	65%
5	Black2	290	1000	11%	89%	76%	-15%	32%	65%
5	Middle3	265	500	13%	77%	67%	-9%	32%	59%
6	Swift1	228	500	15%	-11%	57%	-4%	7%	45%
6	Swift2	244	1000	13%	-16%	65%	-10%	3%	50%
6	Middle2	279	1000	13%	-16%	65%	-10%	3%	50%
6	BASI32	198	200	18%	-5%	46%	4%	13%	38%
6	Swift3	247	1000	13%	-16%	65%	-10%	3%	50%
6	Middle1	247	1000	13%	-16%	65%	-10%	3%	50%
6	BASI51	185	100	21%	-1%	39%	10%	16%	33%
6	BASI53	199	200	18%	-5%	46%	4%	13%	38%
6	BASI55	226	500	15%	-11%	57%	-4%	7%	45%
8	B41a	336	1000	14%	80%	102%	37%	21%	82%
8	B41b	319	1000	14%	80%	102%	37%	21%	82%
8	B41c	327	1000	14%	80%	102%	37%	21%	82%
8	Midd-Swift1	337	1000	14%	80%	102%	37%	21%	82%
8	BASI57	326	1000	14%	80%	102%	37%	21%	82%
8	BASI58	334	1000	14%	80%	102%	37%	21%	82%
8	B59a2	321	1000	14%	80%	102%	37%	21%	82%
8	B59b	313	1000	14%	80%	102%	37%	21%	82%
8	B59c	308	1000	14%	80%	102%	37%	21%	82%
8	B59d	308	1000	14%	80%	102%	37%	21%	82%
8	B60A	300	1000	14%	80%	102%	37%	21%	82%
9	LR4	265	500	22%	70%	67%	43%	23%	55%
9	LR1	235	200	23%	56%	55%	40%	23%	47%
9	B29a	241	200	23%	56%	55%	40%	23%	47%
9	B29b	230	200	23%	56%	55%	40%	23%	47%
9	BASI42	260	500	22%	70%	67%	43%	23%	55%
9	B43a	247	200	23%	56%	55%	40%	23%	47%
9	B43b	290	1000	21%	83%	76%	46%	24%	61%
9	B47a	298	1000	21%	83%	76%	46%	24%	61%
9	BASI56	286	1000	21%	83%	76%	46%	24%	61%
11	B47b	290	200	8%	65%	112%	25%	49%	57%
11	B47c	292	200	8%	65%	112%	25%	49%	57%

11	B60b	290	200	8%	65%	112%	25%	49%	57%
11	B60C	292	200	8%	65%	112%	25%	49%	57%
11	BASI61	283	200	8%	65%	112%	25%	49%	57%
11	B62a	279	200	8%	65%	112%	25%	49%	57%
11	B62b	279	200	8%	65%	112%	25%	49%	57%
11	B62c	256	100	8%	56%	92%	23%	44%	56%
11	B62d	225	100	8%	56%	92%	23%	44%	56%
15	Kinston	213	50	6%	47%	74%	11%	41%	57%
15	B62e	220	50	6%	47%	74%	11%	41%	57%
15	B62f	199	25	6%	40%	59%	10%	37%	55%

Table S4
Percent change (Δ) in precipitation probability statistics from 2025–2054 to 2070–2099 in all subset cells. DD-EDDE cell number corresponds with Jalowska et al. (2021) [14].

**RP (yr)**	**Duration 24h**						**Duration 72h**					
	**C_5**	**C_6**	**C_8**	**C_9**	**C_11**	**C_15**	**C_5**	**C_6**	**C_8**	**C_9**	**C_11**	**C_15**
**CESM 4.5**												
**25**	23	25	18	26	10	6	16	22	24	33	19	10
**50**	21	23	18	25	9	6	10	16	26	35	21	11
**100**	19	21	17	24	8	5	4	10	28	37	23	13
**200**	17	18	16	23	8	4	-2	4	30	40	25	15
**500**	13	15	15	22	6	3	-9	-4	34	43	28	18
**1000**	11	13	14	21	6	2	-15	-10	37	46	31	21
**CESM 8.5**												
**25**	34	7	27	29	41	40	30	23	19	22	35	37
**50**	42	3	35	37	48	47	30	20	20	22	39	41
**100**	51	-1	44	46	56	55	31	16	20	23	44	46
**200**	61	-5	53	56	65	63	31	13	20	23	49	51
**500**	77	-11	68	70	78	76	32	7	21	23	57	59
**1000**	89	-16	80	83	88	87	32	3	21	24	63	65
**GFDL-CM3 8.5**												
**25**	34	26	54	34	60	59	36	24	50	33	52	55
**50**	41	32	61	41	75	74	41	28	55	37	54	57
**100**	48	39	69	48	92	91	46	33	61	42	56	59
**200**	56	46	78	55	112	110	51	38	67	47	57	60
**500**	67	57	91	67	143	141	59	45	75	55	59	62
**1000**	76	65	102	76	170	169	65	50	82	61	60	63

References

1. Spero, T.L.; Bowden, J.B.; Mallard, M.S.; Nolte, C.G.; Willison, J.; Jalowska, A.M.; Talgo, K.D.; Reynolds, L.J.; Brehme, K. EPA Dynamically Downscaled Ensemble (EDDE): Datasets for adaptation and resilience planning for environmental endpoints. *Bull. Am. Meteorol. Soc*. **2025**, *Submitted.*
2. EPA, 2024. EPA Dynamically Downscaled Ensemble (EDDE), Version 1. https://doi.org/10.23719/1530964 (2024).
3. Mearns, L.O.; McGinnis, S.; Daniel, K.; Raymond, A.; Biner, S.; Bukovsky, M.; Chang, H-I; Christensen,O.; Herzmann, D.; Jiao, Y.; Kharin, S.; Lazare, M.; Nikulin, G.; Qian, M.; Scinocca, J.; Winger, K.; Castro, C.; Frigon, A.; Gutowski, W. The NA-CORDEX dataset, version 1.0. https://doi.org/10.5065/D6SJ1JCH (2017). Accessed (15 February 2021).
4. Pierce, D.W.; Cayan D.R.; Thrasher B.L. Statistical downscaling using Localized Constructed Analogs (LOCA). J. Hydrometeorol. **2014**, 15, 2558–2585.
5. 5 Abatzoglou, J.T.; Brown, T.J. A comparison of statistical downscaling methods suited for wildfire applications. Int. J. Climatol. **2012**, 32(5), 772–780.
6. Abatzoglou, J.T. Development of gridded surface meteorological data for ecological applications and modelling. Int. J. Climatol. **2013**, 33(1), 121–131.
7. Skamarock, W.C.; Klemp, J.B. A time-split nonhydrostatic atmospheric model for weather research and forecasting applications. *J. Comput. Phys*. **2008**, *227*, 3465–3485.
8. Herwehe, J. A., Alapaty, K., Spero, T. L., Nolte, C. G. Increasing the credibility of regional climate simulations by introducing subgrid-scale cloud-radiation interactions. *J. Geophys. Res. Atmos.* **2014**, 119, 5317–5330.
9. Otte, T.L.; Nolte, C.G.; Otte, M. J.; Bowden, J. H. Does nudging squelch the extremes in regional climate modeling? *J. Clim*. **2012** *25*, 7046–7066.
10. Mallard, M.S.; Nolte, C.G.; Bullock, O.R.; Spero, T.L.; Gula, J. Using a coupled lake model with WRF for dynamical downscaling. *J. Geophys. Res. Atmos*. **2014**, *119(12)*, 7193–7208.
11. Spero, T.L.; Nolte, C.G.; Bowden, J.H.; Mallard, M.S.; Herwehe, J.A. The impact of incongruous lake temperatures on regional climate extremes downscaled from the CMIP5 archive using the WRF model. *J. Clim*. **2016**, *29*, 839–853.
12. NCDOT NCEM (North Carolina Department of Transportation and North Carolina Division of Emergency Management). *Neuse River Basin Flood Analysis and Mitigation Strategies Study*. https://files.nc.gov/rebuildnc/documents/files/neuse_mitigation_report.pdf. (NC Department of Transportation, 2018).
13. Bonnin, G.M.; Martin, D.; Lin, B.; Parzybok, T.; Yekta, M.; Riley, D. NOAA Atlas 14, Volume 2, Version 3.0, Precipitation Frequency Atlas of the United States. NOAA, National Weather Service, **2006**.
14. Jalowska, A.M.; Spero, T.L.; Bowden, J.H. Projecting changes in extreme rainfall from three tropical cyclones using the design-rainfall approach. *npj Clim. Atmos. Sci*. **2021**, *4*, 23.
